# Nephrotoxicity of New Antibiotics: A Systematic Review

**DOI:** 10.3390/toxics13070606

**Published:** 2025-07-19

**Authors:** Panagiotis Stathopoulos, Laura T. Romanos, Charalampos Loutradis, Matthew E. Falagas

**Affiliations:** 1 Alfa Institute of Biomedical Sciences, 9 Neapoleos Street, Marousi, 151 23 Athens, Greece; 2 School of Medicine, European University Cyprus, 6 Diogenous Str., Egkomi, Nicosia 2404, Cyprus; loutradis_haris@hotmail.com; 3 First Department of Urology, School of Medicine, Faculty of Health Sciences, Aristotle University of Thessaloniki, 541 24 Thessaloniki, Greece; 4 Department of Medicine, Tufts University School of Medicine, 145 Harrison Ave, Boston, MA 02111, USA

**Keywords:** aztreonam/avibactam, cefepime/enmetazobactam, cefiderocol, ceftobiprole, contezolid, gepotidacin, imipenem/cilastatin/relebactam, lascufloxacin, lefamulin, levonadifloxacin, plazomicin, sulbactam/durlobactam

## Abstract

Drug-induced nephrotoxicity is a common and serious problem in clinical practice. We conducted a systematic review of studies reporting nephrotoxicity events associated with antibiotics approved since 2018. The agents assessed included aztreonam/avibactam, cefepime/enmetazobactam, cefiderocol, ceftobiprole, contezolid, gepotidacin, imipenem/cilastatin/relebactam, lascufloxacin, lefamulin, levonadifloxacin, plazomicin, and sulbactam/durlobactam. Literature searches were conducted in PubMed, Scopus, Web of Science, and major pharmacovigilance databases (Vigibase, FAERS, EudraVigilance, EMA, FDA, NMPA, PMDA, and CDSCO) in May 2025, along with reference citation tracking. Studies were included if they reported safety or adverse event data. The risk of bias was assessed using validated tools in accordance with the study design. Out of 2105 potentially relevant records, 74 studies met inclusion criteria, comprising 52 clinical trials, 17 observational studies, 1 registry-based study, 3 case series, and 1 case report. Nephrotoxicity was rarely reported for any of the newly approved antibiotics. No renal adverse events were found in the available studies for aztreonam/avibactam, levonadifloxacin, and contezolid. Most studies were of moderate to high quality; two were classified as low quality. However, nephrotoxicity was inconsistently assessed, with variable definitions and methodologies used. Although current data suggest a low frequency of nephrotoxicity, limitations in study design and reporting preclude firm conclusions. There is a need for post-marketing studies to better characterize renal safety. Clinicians should remain vigilant and continue to monitor for and report renal-related adverse events.

## 1. Introduction

Since the inception of therapeutics, medical practice has relied on the administration of exogenous substances, initially herbs and foods, and, more recently, pharmaceutical agents, to alter physiological responses and improve disease outcomes [1]. However, it has long been recognized that any therapeutic agent may also cause harm through adverse drug reactions (ADRs). Modern drug development aims to quantify both efficacy and safety before a drug reaches clinical use, with toxicity being a common reason for discontinuation in early or late development phases. Even after approval, newly marketed drugs undergo close post-marketing surveillance, as pre-approval trials often lack the power to detect rare or delayed adverse events [2,3]. Drug toxicity is, thus, a common cause of discontinuation in the drug development pipeline [3].

Among the most serious ADRs is drug-induced nephrotoxicity, which can lead to acute kidney injury (AKI) and represents one of the most common severe adverse events [4,5,6]. Up to 60% of AKI events in hospitalized patients are drug-induced, and the relevant morbidity and mortality are also high [4]. Antibiotics are frequently implicated in nephrotoxicity events [4]. They may affect renal function through diverse mechanisms, including direct tubular toxicity, altered hemodynamics, crystal nephropathy, and hypersensitivity reactions such as acute interstitial nephritis [7,8]. Aminoglycosides, vancomycin, and beta-lactams are well-known examples of nephrotoxic antimicrobials [9].

Over the past decade, due to the growing crisis of antimicrobial resistance, several new antibiotics have been introduced into clinical practice [10]. Meanwhile, pharmacovigilance databases such as FAERS and EudraVigilance have begun to collect real-world reports of renal adverse events linked to these newer antibiotics; however, systematic assessments are lacking [11]. These reporting systems offer valuable insight into potential safety concerns under routine clinical conditions. This highlights the importance of robust, comprehensive safety evaluations of newly approved antibiotics [12,13].

In this context, we conducted a systematic review of published studies reporting nephrotoxicity associated with antibiotics approved since 2018. We did not include antiviral, antifungal, anti-mycobacterial, and antiparasitic drugs. The antibiotics assessed were, in alphabetical order, aztreonam/avibactam (AbbVie Inc., North Chicago, IL, USA), cefepime/enmetazobactam (Allecra Therapeutics GmbH, Weil am Rhein, Germany), cefiderocol (Shionogi & Co., Ltd., Osaka, Japan), ceftobiprole (Basilea Pharmaceutica Ltd., Basel, Switzerland), contezolid (MicuRx Pharmaceuticals, Hayward, CA, USA), gepotidacin (GlaxoSmithKline plc, Brentford, UK), imipenem/cilastatin/relebactam (Merck & Co., Inc., Rahway, NJ, USA), lascufloxacin (Kyorin Pharmaceutical Co., Ltd., Tokyo, Japan), lefamulin (Nabriva Therapeutics, Dublin, Ireland), levonadifloxacin (Wockhardt Ltd., Mumbai, India), plazomicin (Achaogen Inc., South San Francisco, CA, USA), and sulbactam/durlobactam (Innoviva Specialty Therapeutics, Morrisville, NC, USA). This systematic review aimed to evaluate the frequency and characteristics of nephrotoxicity reported in published clinical studies involving the antibiotics above, as well as real-world data from pharmacovigilance databases.

## 2. Methods

### 2.1. Data Sources and Eligibility Criteria

We conducted a systematic review in accordance with the Preferred Reporting Items for Systematic Reviews and Meta-Analyses (PRISMA) 2020 guidelines. The research protocol was not registered in a systematic review database. The databases we searched were PubMed, Scopus, and Web of Science from their inception to May 2025. We searched for studies on humans, encompassing randomized controlled trials (RCTs) and observational studies, that assessed the safety, adverse events, and nephrotoxicity of each of the antibiotics mentioned above separately, as either a primary or secondary outcome. We excluded studies if they did not explicitly assess the safety of the drug, involved animal subjects, were published in languages other than English, or were limited to conference abstracts. We also searched for reports of nephrotoxicity in pharmacovigilance databases (VigiBase, FAERS, EudraVigilance) and regulatory agencies (FDA, EMA, NMPA, PMDA, CDSCO) in June 2025.

### 2.2. Search Strategy

The search strategy is presented in Supplementary File S1. We used search terms such as “safety”, “adverse events”, “adverse reactions”, “nephrotoxicity”, “renal impairment”, and “kidney injury”. The search strings were tailored to each database and conducted separately for each antibiotic. We also manually searched relevant reviews to identify additional studies that may have been missed in the initial search.

### 2.3. Screening and Data Collection Process

Deduplication of studies retrieved from different databases was performed with the automatic deduplication of the Rayyan tool (https://www.rayyan.ai/ accessed on 1 May 2025). One reviewer (PS) independently screened all records retrieved from the search strategy, initially from the title and abstract with the Rayyan tool, and then retrieved and screened full texts. Studies were considered eligible if they presented data on the condition for which the drug was used, the route of administration, adverse events, or laboratory abnormalities.

Cases with nephrotoxicity in our analysis were based on the definitions reported by the authors of the studies. The definition of nephrotoxicity could have included dysregulation of kidney function, such as AKI, interstitial nephritis, and tubular necrosis, as well as an increase in serum creatinine levels without necessarily meeting the KDIGO criteria [14] and proteinuria. Studies presenting data on pharmacokinetics, along with safety, were also considered eligible. The same reviewer (PS) extracted data from all eligible studies, and a second reviewer (LTR) verified the accuracy of the extracted data. Disputes were settled by consensus with a senior author (MEF). Extracted data included the type of study, participants’ health status, the proportion of nephrotoxicity events, and the specific manifestation of nephrotoxicity. The data were organized into categories following a predefined methodological framework.

### 2.4. Risk of Bias Assessment

The quality of the studies included was assessed using risk-of-bias tools. The Jadad scale was used for RCTs, the ROBINS-I v2 for non-RCTs, and the Newcastle–Ottawa scale for observational studies. Pharmacokinetics and small Phase 1 studies were not assessed for risk of bias due to inherent methodological limitations.

### 2.5. Data Synthesis

Due to the heterogeneity in study design and outcomes, a statistical analysis was deemed inappropriate. We chose to assess the data extracted on the potential nephrotoxicity of each antibiotic without using statistical techniques of meta-analysis.

### 2.6. Adherence to PRISMA Guidelines

Adherence to PRISMA guidelines is reported in the relevant checklist in Supplementary File S2.

## 3. Results

### 3.1. Search Results

**Figure 1:**
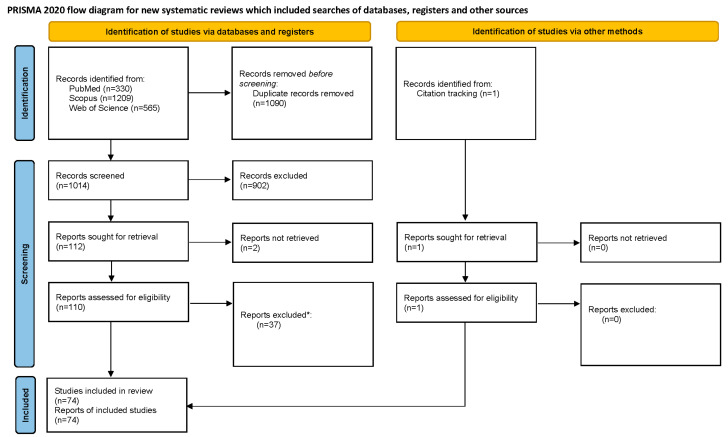
PRISMA 2020 flow diagram for new systematic reviews that included searches of databases, registers, and other sources. * Reasons for exclusion are presented in Supplementary Table S1.

Our search strategy identified 2105 studies. The automatic deduplication tool identified 1090 duplicate records. We screened 1014 potentially relevant studies, and after screening, we extracted data from 74 studies that were ultimately included in the analysis. The whole process of identification, screening, retrieval, and inclusion of relevant studies is presented as a flow diagram in Figure 1. The studies we excluded after full-text screening are given in Supplementary Table S1 [15,16,17,18,19,20,21,22,23,24,25,26,27,28,29,30,31,32,33,34,35,36,37,38,39,40,41,42,43,44,45,46,47,48,49,50,51].

Table 1 presents the main characteristics of the 12 antibiotics approved from 2018 to the time of writing, including the generic and trade names, year and region of approval, chemical structure, antibiotic class, and indications. Table 2 presents data on the included studies (clinical trials, cohort studies, case series, and case reports) and the reported effect of each antibiotic on renal function, as well as data from pharmacovigilance databases and regulatory agencies in countries where each antibiotic is marketed. The studies are organized by the antibiotic used and presented in chronological order.

### 3.2. β-Lactams and β-Lactam/β-Lactamase Inhibitor Combination Antibiotics

For aztreonam/avibactam, three clinical trials [52,53,54] were identified. None of the studies reported renal-related adverse events.

For cefepime/enmetazobactam, two clinical trials were identified [55,56]. In a Phase 3 trial involving patients with complicated urinary tract infections or acute pyelonephritis, renal and urinary adverse events were reported in 22/516 (4.3%) patients [56]. However, the specific nature of these events was not specified. The other trial did not report renal-related adverse events.

For cefiderocol, five clinical trials [57,58,59,60,61], one prospective study [62], five retrospective cohort studies [63,64,65,66,67], one case report [68], and one case series [69] were identified. In a Phase 2 trial on pediatric patients with bacterial infections [61], renal impairment was reported in 2/29 (6.9%) patients receiving cefiderocol. Acute interstitial nephritis attributed to cefiderocol was reported in 1/244 (0.4%) patients in a retrospective cohort study of patients receiving cefiderocol for Gram-negative infections [66]. The case report also described acute interstitial nephritis associated with cefiderocol [68]. None of the other studies reported renal-related adverse events.

For ceftobiprole, eight clinical trials [70,71,72,73,74,75,76,77], five retrospective cohort studies [78,79,80,81,82], and one cross-sectional, survey-based registry study [83] were identified. In a Phase 3 clinical trial, renal-related adverse events were reported in 10/543 (1.9%) patients with complicated skin and skin structure infections [71]. In another Phase 3 trial [72], 1/348 (0.3%) patients with complicated skin and skin structure infections experienced an increase in serum creatinine that was twice the upper normal limit. In a retrospective cohort study of 396 patients receiving ceftobiprole for bacterial infections, AKI was reported in 35 patients [82]. More specifically, 9/46 (19.6%) patients receiving high-dose ceftobiprole developed an AKI, which was attributed to the drug in two patients. The same event was reported in 26/350 (7.4%) patients receiving the recommended dose, but the association with the drug was not specified. In the cross-sectional registry study, one patient developed interstitial nephritis, but it was considered unrelated to the drug [83]. None of the other studies reported renal-related adverse events.

For imipenem/cilastatin/relebactam, six clinical trials [98,99,100,101,102,103], one retrospective cohort study [104], and one case series [105] were identified. In a Phase 2 trial of patients with complicated urinary tract infections, proteinuria was reported in 1/99 (1.1%) patients [98]. In a Phase 3 trial of patients with infections caused by imipenem-resistant bacteria, AKI was reported in 3/29 (10.3%) patients [101]. The retrospective study involved patients with Gram-negative infections and reported AKI in 1/106 (0.9%) patients [104]. None of the other studies reported renal-related adverse events.

For sulbactam/durlobactam, two clinical trials were identified [124,125]. In a Phase 3 trial of patients with infections due to *A. baumannii-calcoaceticus* complex bacteria, AKI was reported in 12/91 (13.2%) patients [125]. The other trial did not report any renal-related adverse events.

### 3.3. Fluoroquinolones

For lascufloxacin, three trials [106,107,108] and one retrospective cohort study [109] were identified. In a Phase 1 trial of healthy subjects, an isolated increase in serum creatinine was reported in 2/77 (2.6%) participants [106]. In a Phase 3 trial of patients with community-acquired pneumonia, AKI was reported in 1/114 (0.9%) patients [108]. None of the other studies reported renal-related adverse events.

For levonadifloxacin, two retrospective cohort studies [116,117] and two prospective cohort studies [118,119] were identified. None of the studies reported renal-related adverse events.

### 3.4. Other Drugs

For contezolid, five clinical trials [84,86,87,88,126] and a case series [89] were identified. None of the studies reported renal-related adverse events.

For gepotidacin, eight clinical trials [90,91,92,93,94,95,96,97] were identified. In a Phase 1 trial of healthy adults and elderly subjects, proteinuria was reported in 48/72 (66.7%) adults aged 18 to 60 years receiving repeated ascending oral doses and in 4/29 (13.8%) participants aged more than 65 years receiving repeated oral doses of gepotidacin [94].

For lefamulin, six clinical trials [110,111,112,113,114,115] were identified. In a Phase 1 trial of healthy subjects, AKI was reported in 2/20 (5%) patients [115]. None of the other studies reported renal-related adverse events.

For plazomicin, four clinical trials [120,121,122,123] were identified. In a Phase 2 trial of patients with complicated urinary tract infections and acute pyelonephritis, 7/85 (2.1%) patients had renal-related adverse events or laboratory abnormalities [121]. More specifically, one patient developed an AKI, one patient had mild azotemia, and five patients had a 0.5 mg/dL increase in serum creatinine from baseline, but it was not reported as an AKI. In a Phase 3 trial of patients with complicated urinary tract infections, it was reported that 11/303 (3.6%) patients experienced deteriorating renal function, and 21/303 (6.9%) patients had an increase in serum creatinine equal to or greater than 0.5 mg/dL. Still, it was also not reported as an AKI [122].

### 3.5. Risk of Bias Assessment

The risk of bias assessment is presented in Supplementary Tables S2–S4. The studies that were not assessed for risk of bias are presented, along with the reasoning, in Supplementary Table S5. We assessed 27 clinical trials using the Jadad scale, 5 non-randomized clinical trials using the ROBINS-I v2 tool, and 16 cohort studies using the Newcastle–Ottawa scale. Additionally, 26 studies were not assessed using risk-of-bias tools. Of the 27 clinical trials, 19 were evaluated as high-quality [56,58,59,71,72,73,76,77,87,90,96,98,101,102,103,111,112,121,122] and 8 as moderate-quality studies [53,54,60,74,91,97,110,125]. Of the five non-randomized clinical trials, three were at low risk [52,61,93], one was at moderate risk [107], and one was at high risk of bias [108]. Of the 17 cohort studies, 5 were high-quality [79,81,82], 10 were moderate-quality [64,65,66,78,104,109,116,117,118,119], and 1 was low-quality [80].

## 4. Discussion

### 4.1. Summary and Interpretation of the Results

Our review included 74 studies that addressed the safety of antibiotics and provided data on their potential nephrotoxicity. There was a rare manifestation of nephrotoxicity for cefepime/enmetazobactam, cefiderocol, ceftobiprole, gepotidacin, imipenem/cilastatin/relebactam, lascufloxacin, lefamulin, plazomicin, and sulbactam/durlobactam. We did not identify any study reporting nephrotoxicity of aztreonam/avibactam, levonadifloxacin, and contezolid.

The only antibiotic with a relevant precaution for nephrotoxicity, as per regulatory authorities, is plazomicin. In our study, we identified a Phase 3 trial reporting a relatively high proportion (10.5%) of patients with complicated urinary tract infections, with affected renal function associated with the use of plazomicin [122]. The Phase 2 trial of patients with the same condition also reported nephrotoxicity events, with a smaller proportion (2.1%) [121]. The overall health status, aside from the infection, of the patients with plazomicin-associated nephrotoxicity included in those studies is not reported. However, nephrotoxicity was not reported in the two Phase 1 trials of healthy subjects included in our analysis. This observation could suggest that either nephrotoxicity is more common in patients with urinary tract infections or that healthy subjects or subjects with little comorbidity are less prone to nephrotoxicity events.

A relatively high proportion of patients with *A. baumannii* infections treated with the sulbactam/durlobactam combination were complicated by acute kidney injury events (13.2%), as reported in a Phase 3 trial [125]. In the same pattern as for plazomicin, a Phase 1 study of sulbactam/durlobactam, involving 34 healthy subjects and, notably, subjects with chronic kidney disease, did not report any nephrotoxicity events. For this drug, nephrotoxicity is listed as an adverse reaction.

Our results are consistent with the existing safety profiles and guidelines provided by the regulatory authorities responsible for the surveillance of each drug. Notably, all antibiotics for which nephrotoxicity was observed in our analysis are also noted by regulatory bodies with warnings or precautions for this adverse event. Even for agents without or rarely reported nephrotoxicity in the included studies, regulatory authorities list nephrotoxicity as a potential adverse event, underscoring the importance of vigilance in its prevention and early detection.

This discrepancy can be attributed to several factors. First, clinical trials often involve carefully selected patient populations and may exclude those with risk factors for renal injury (e.g., pre-existing CKD, polypharmacy, or critical illness). In addition, most trials have a small number of patients and short follow-up periods and thus are not designed to detect rare or late-onset nephrotoxicity. Also, definitions of nephrotoxicity vary across studies and are not reported consistently, ranging from mild creatinine elevations to a clinically apparent AKI. In contrast, post-marketing surveillance systems present data from real-world patients. Overall, these observations underscore the crucial role of post-marketing data in complementing clinical trial data, particularly for safety outcomes such as nephrotoxicity, which are not always well-defined before drug approval.

### 4.2. Strengths and Limitations

This is the first systematic review to evaluate the nephrotoxicity of the recently approved antibiotics. Notably, it included 12 new antibiotics that are currently in use. Our systematic review was conducted in accordance with the PRISMA guidelines, ensuring its methodological soundness. An extensive search across three different databases yielded a large number of potentially relevant studies. Our search strategy was comprehensive and not limited to database searching alone. We utilized specialized software tools to expedite and enhance the screening process. Additionally, we manually searched the citations of potentially relevant studies for our review to ensure that we did not miss any eligible studies.

An inherent limitation of our study is that nephrotoxicity is a broad term encompassing multiple, often indistinct pathophysiological processes. Consequently, it is possible that some of the studies we included that reported no nephrotoxicity may have had subclinical or undetected renal toxicity that was overlooked. This can become apparent in post-marketing surveillance, where drugs are administered to patients with considerable comorbidity, which may predispose patients to have such adverse reactions. Notably, chronic kidney disease (CKD) patients, who may be more susceptible to nephrotoxic effects, are frequently excluded from clinical trials, further limiting the generalizability of the findings to this complicated population.

### 4.3. Scarcity of Post-Marketing Published Studies on Safety

The nephrotoxicity of new antimicrobials or any other new drugs noted in clinical trials is expected to be less compared to real clinical practice. This is partially explained by the considerable number of inclusion and exclusion criteria in RCTs, in which patients with existing risk factors for AKI are commonly excluded. Although this approach improves the methodological rigor of RCTs and may offer a better understanding of the comparative effectiveness of the newly studied drug, it suffers regarding the issue of the generalizability of the study’s results. This is the reason for the current movement toward pragmatic trials, as opposed to traditional RCTs with numerous inclusion and exclusion criteria [127].

Higher drug-related toxicity has been observed in post-marketing studies compared to RCTs. Several new drugs were withdrawn from the market after approval from regulatory organizations due to emerging reports on adverse events in real-world data. They include a considerable number of antimicrobial agents, including fluoroquinolones such as trovafloxacin, temafloxacin, and grepafloxacin [128,129,130].

The pathophysiology of drug-induced nephrotoxicity is complex, and patients may have many contributing factors predisposing them to such events. It occurs more frequently in patients with previous renal dysfunction, comorbidity from other systems and organs, and co-administration of other drugs with potential nephrotoxicity. For example, the co-administration of an aminoglycoside or polymyxin with a new antibiotic may increase the likelihood of nephrotoxicity development compared to the proportions when each agent is administered separately [131,132].

Also, modern meticulous management of abnormalities of the hydration status, electrolyte imbalances, and other types of comorbidity helps decrease the drug-induced toxicity, including nephrotoxicity [133,134]. This has been demonstrated by a reduced frequency of toxicity of colistin, a polymyxin antibiotic, in recent studies compared to previous studies [135,136]. Dehydration contributes significantly to the pathophysiologic mechanisms of drug-induced nephrotoxicity. Thus, physicians should be aware of this factor that may increase the likelihood of nephrotoxicity development in clinical practice, especially in critically ill patients.

In addition, nephrotoxicity may exist unnoticed in patients without such contributing factors. Currently, screening for nephrotoxicity in the clinical setting is based solely on basic laboratory tests, including serum urea and creatinine, as well as the calculated glomerular filtration rate. Additionally, urine sample tests may reveal abnormal findings. However, these tests detect nephrotoxicity only when it is already established. Drug-induced nephrotoxicity is a complex process comprising multiple interconnected mechanisms. Data from primary research suggest that there may be biomarkers that can help detect evolving nephrotoxicity before renal dysfunction occurs. Incorporating such tests can improve screening for all patients, specifically those with risk factors for nephrotoxicity, preventing renal-related adverse events [85,137,138].

## 5. Conclusions

The evaluation of the available data suggests that there is a risk of nephrotoxicity associated with each of the antibiotics we studied. However, the scarcity of published data does not allow for the derivation of safe conclusions on the true frequency and type of nephrotoxicity associated with new antibiotics. Therefore, future research should focus on post-marketing surveillance studies that report adverse events in patients encountered in daily clinical practice. Subsequently, clinicians should report their observations on adverse events, including nephrotoxicity, of new antimicrobial agents and other drugs to the relevant national organizations that collect post-marketing data on adverse events.

## Figures and Tables

**Table 1:** Newly approved antibiotics.

Drug (Brand Name)	Year of Approval, Country/Region	Chemical Structure	Class	Indications
**Gepotidacin** **(Blujepa)**	FDA 2025	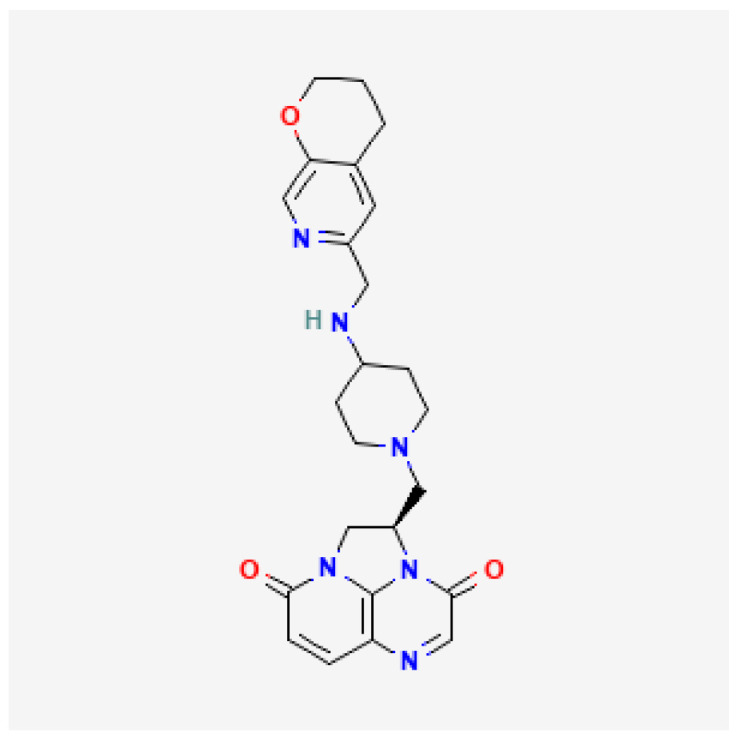	DNA topoisomerase II and IV inhibitor	Uncomplicated UTIs
**Aztreonam/avibactam** ** (Emblaveo)**	FDA 2025, EMA 2024, UK 2024	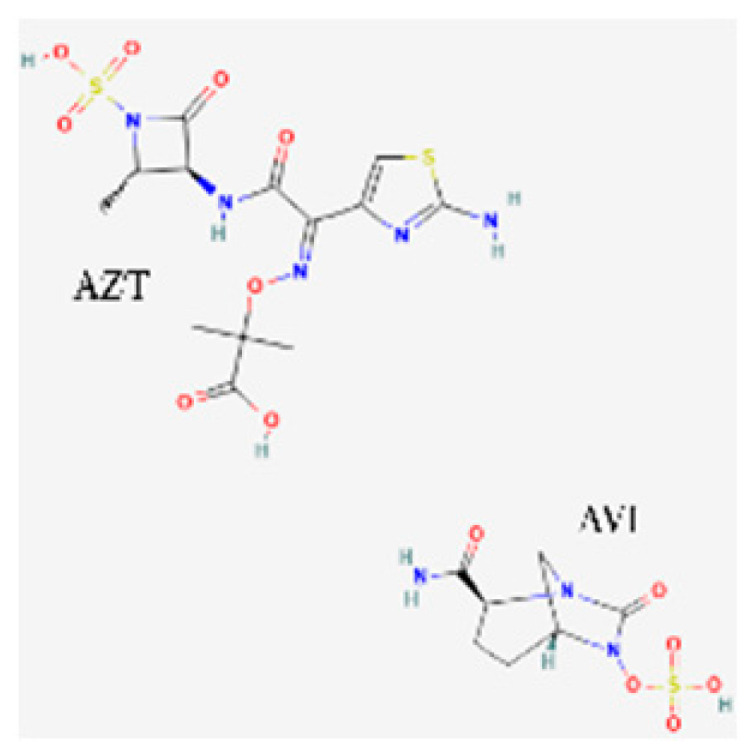	BL/BLI	cIAIs, HAP, VAP, cUTIs
**Cefepime/enmetazobactam** **(Exblifep)**	EMA 2024, FDA 2024	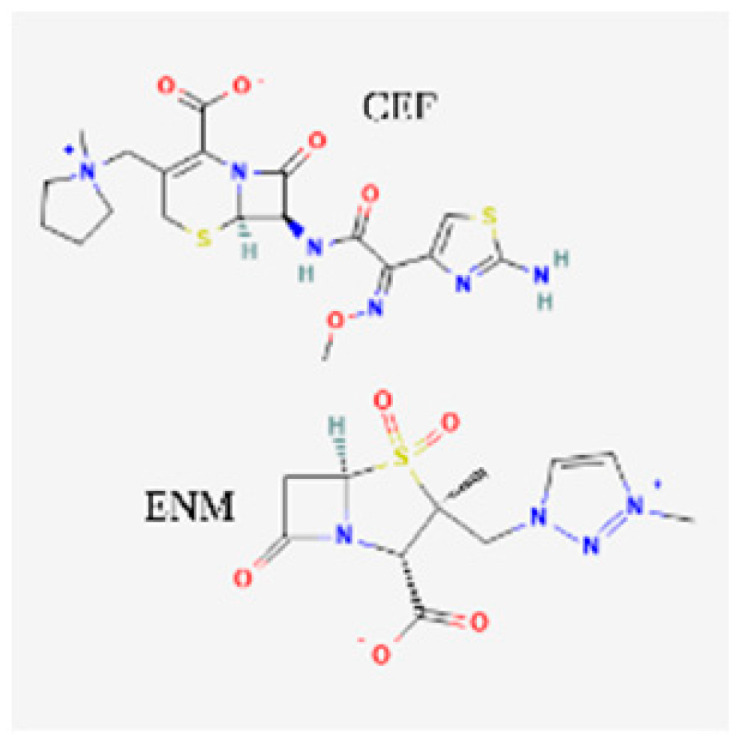	BL/BLI	cUTIs, HAP, VAP
**Ceftobiprole** **(Zevtera)**	FDA 2024	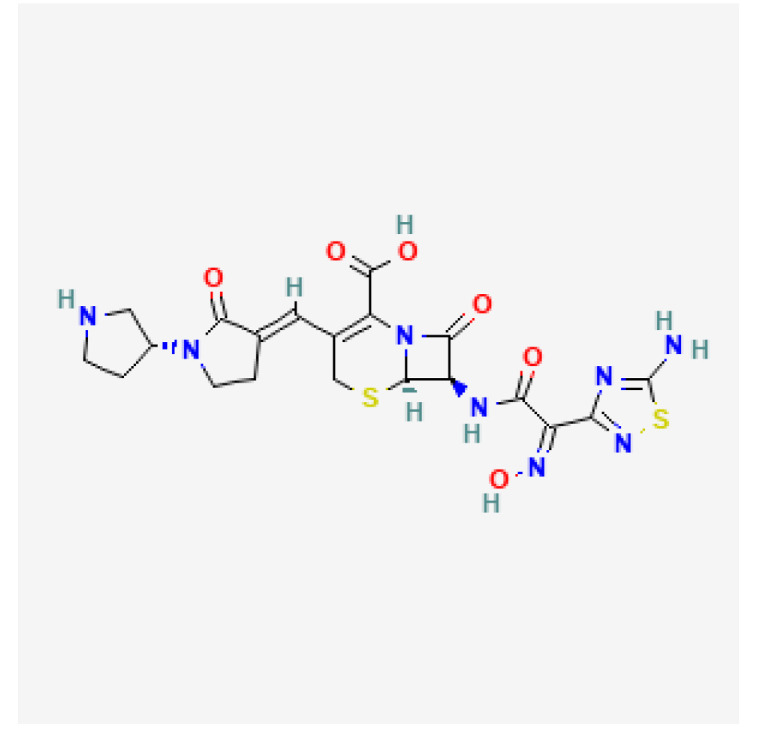	5th gen cephalosporin	HAP, VAP, CAP, IE, ABSSSI
**Levonadifloxacin** **(Emrok)**	NMPA 2024	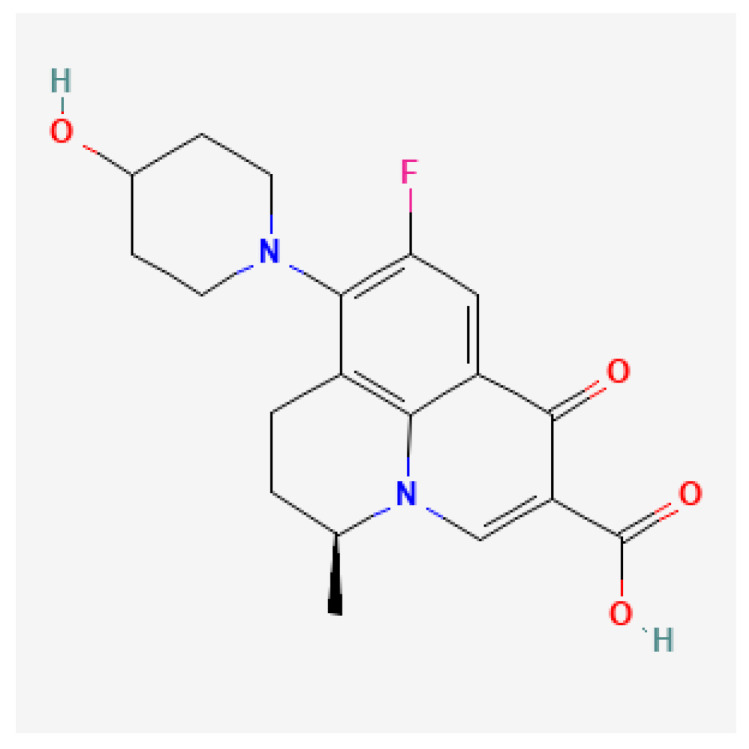	Fluoroquinolone	ABSSSI
**Sulbactam/durlobactam** **(Xacduro)**	FDA 2023	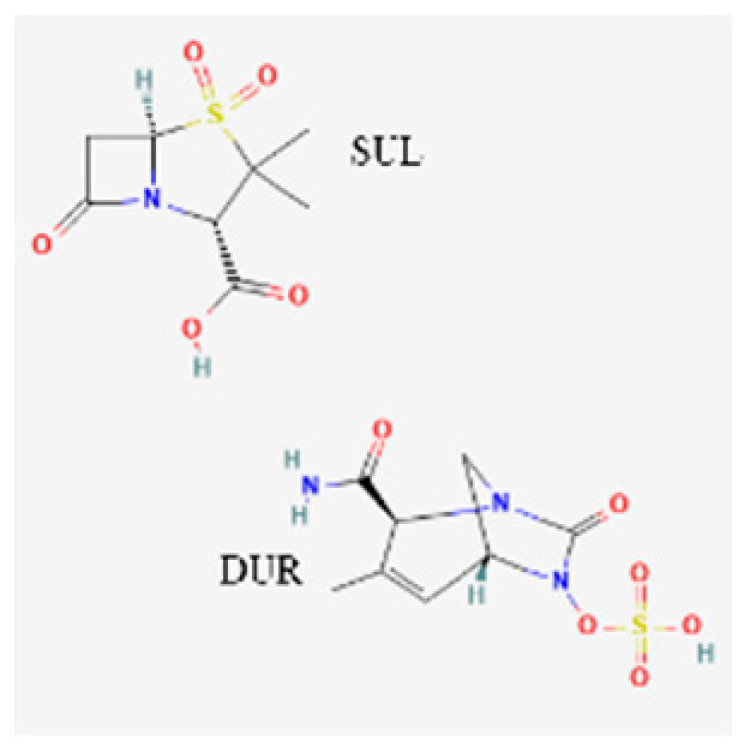	BL/BLI	HAP, VAP
**Contezolid** **(Youxitai)**	NMPA 2021	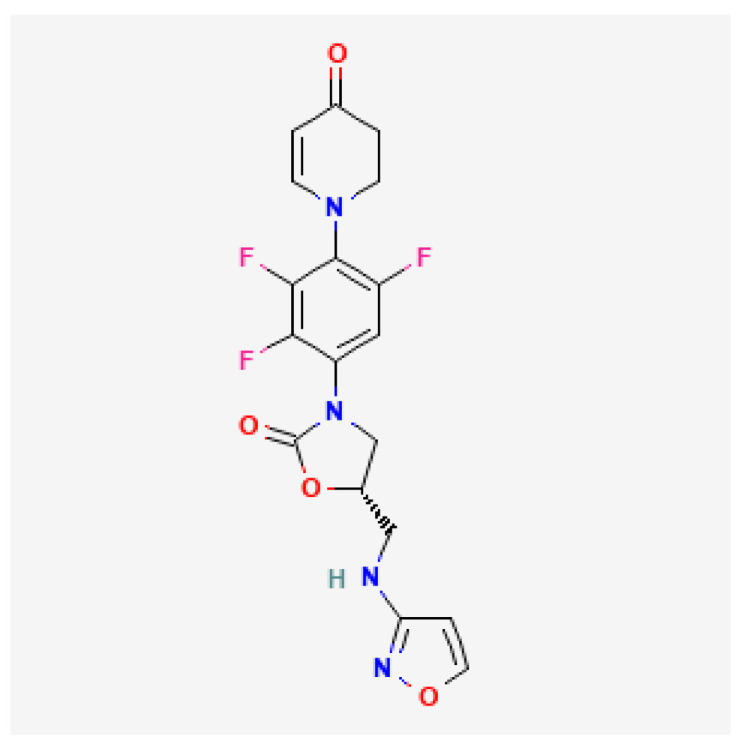	Oxazolidinone	cSSTI
**Cefiderocol** (Fetcroja, Fetroja *)	EMA 2020, FDA 2020	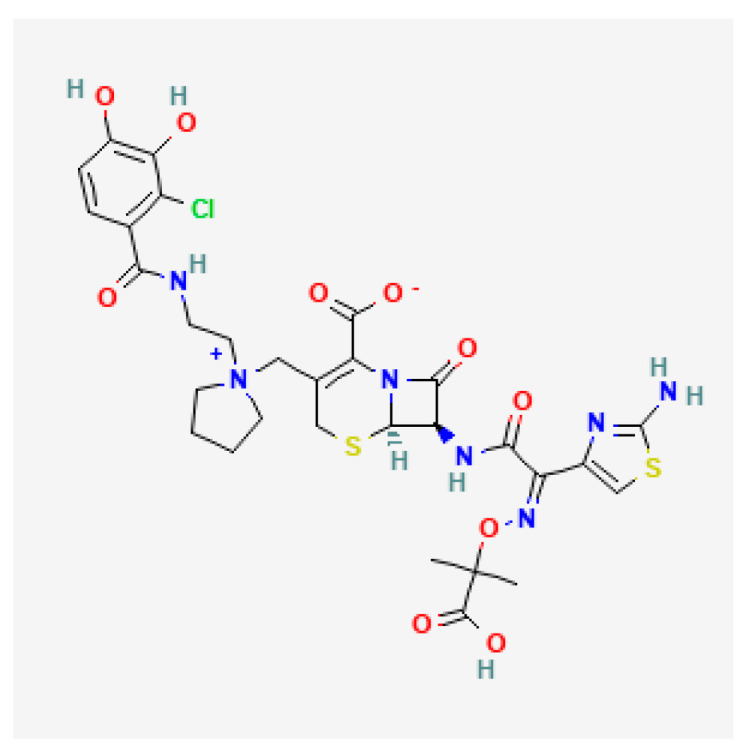	Siderophore cephalosporin	cUTI, HAP, VABP
**Imipenem/cilastatin/relebactam** **(Recarbrio)**	EMA 2020, FDA 2019	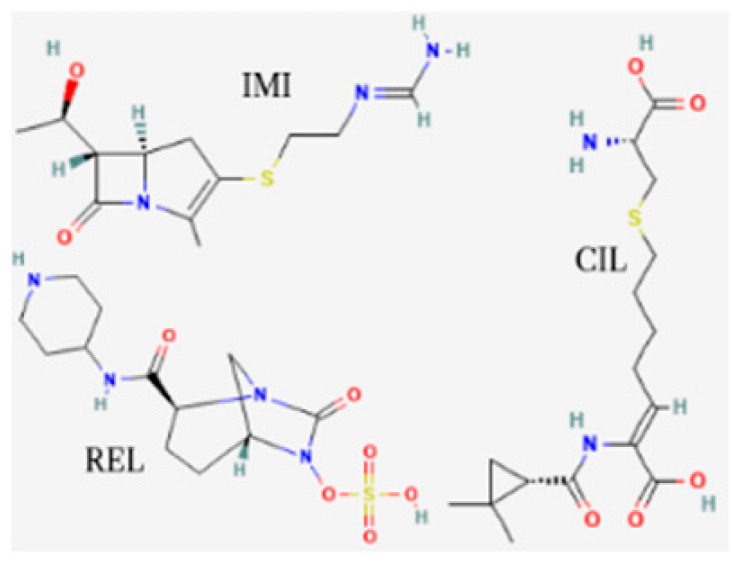	BL/BLI	HABP, VABP, cUTI, cIAI
**Lefamulin** **(Xenleta)**	EMA 2020, FDA 2019	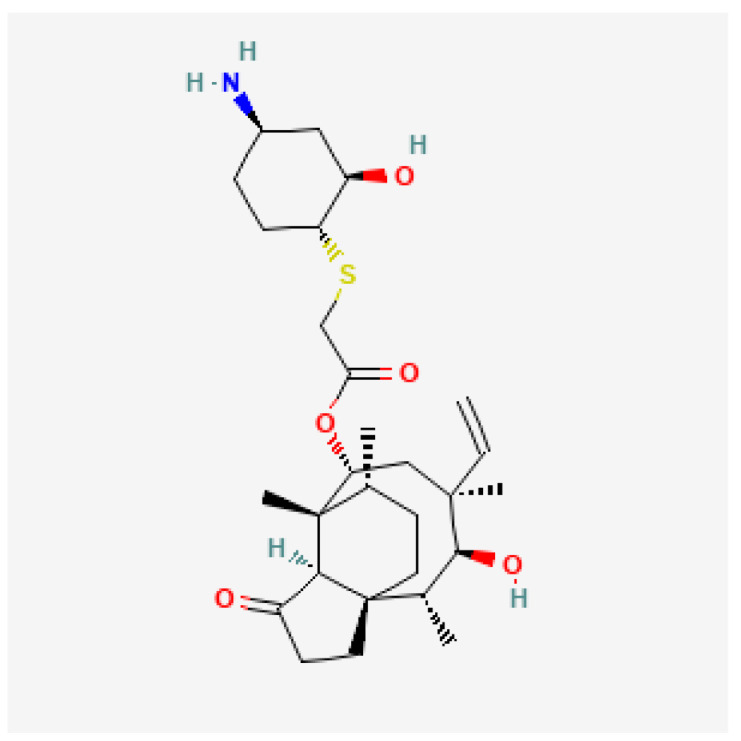	Pleuromutilin, protein synthesis inhibitor	CAP, ABSSSI
**Lascufloxacin** ** (Lasvic)**	Japan 2019	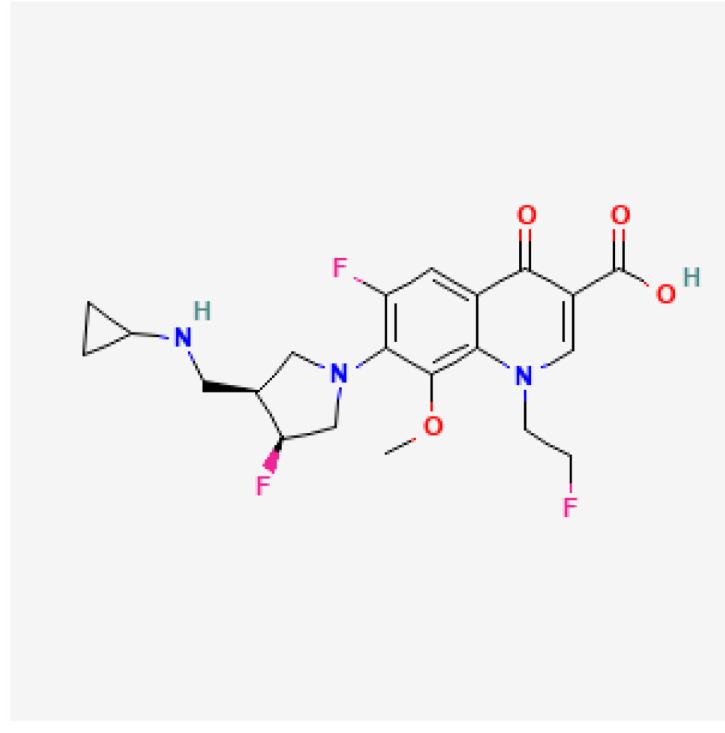	Fluoroquinolone	CAP, RTIs
**Plazomicin** **(Zemdri)**	FDA 2018	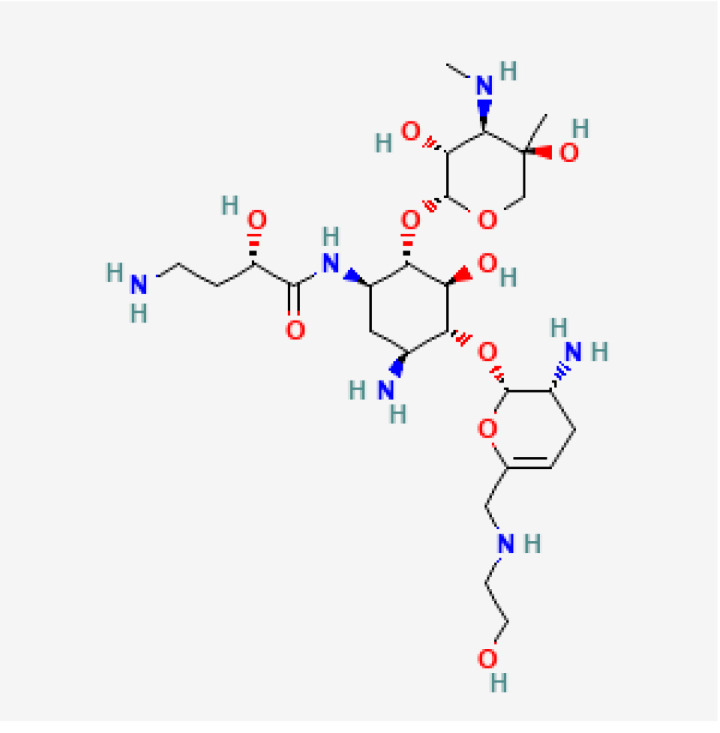	Aminoglycoside	cUTIs

**Abbreviations:** ABSSSI: acute bacterial skin and skin structure infection, AVI: avibactam, AZT: aztreonam, CAP: community-acquired pneumonia, CEF: cefepime, cIAI: complicated intra-abdominal infection, CIL: cilastatin, cSSTI: complicated skin and soft tissue infection, cUTI: complicated urinary tract infection, DUR: durlobactam, ENM: enmatazobactam, HABP: hospital-acquired bacterial pneumonia, HAP: hospital-acquired pneumonia, IE: infectious endocarditis, IMI: imipenem, REL: relebactam, RTI: respiratory tract infection, SUL: sulbactam, UTI: urinary tract infection, VABP: ventilator-associated bacterial pneumonia, VAP: ventilator-associated pneumonia. * The proprietary name for this medication is Fetcroja in the European Union and Fetroja in the United States.

**Table 2:** Nephrotoxicity of new antibiotics in clinical trials, cohort studies, case series, and case reports *.

Author, Year	Ref.	Study Type	Specific Infection or Healthy Subjects	Nephrotoxicity Reported n/N (%)	Manifestation of Nephrotoxicity	Pharmacovigilance and Prescribing Label Data on Nephrotoxicity
*Aztreonam/avibactam*						VigiBase: NR FAERS: NR EudraVigilance: NR EMA: Uncommon FDA: NL
Cornely, 2020	[52]	Phase 2a trial	cIAIs	0/34 (0)		
Carmeli, 2025	[53]	Phase 3 trial	Gram (−) infections	0/275 (0)		
ASSEMBLE study ^a^	[54]	Phase 3 trial	Gram (−) infections	0/12 (0)		
*Cefepime/enmetazobactam*						VigiBase: NR FAERS: NR EudraVigilance: NR EMA: Uncommon FDA: Less than 1%
Das, 2020	[55]	Phase 1 trial	Healthy subjects	0/20 (0)		
Kaye, 2022	[56]	Phase 3 trial	cUTIs or acute pyelonephritis	22/516 (4.3)	Kidney and urinary adverse events, not explicitly specified.	
*Cefiderocol*						VigiBase: 40/500 (8%) reports FAERS: 29 AKI, 9 renal impairment, 3 blood creatinine increase, 2 renal failure, 2 GFR decrease, and 2 renal tubular disorder out of 522 reports EudraVigilance: 30/197 (15.2%) reports EMA: NK FDA: Reported in ≤4% of patients in clinical trials
Saisho, 2018	[57]	Phase 1 trial	Healthy subjects	0/16 (0)		
Portsmouth, 2018	[58]	Phase 2 trial	cUTIs	0/300 (0)		
Wunderink, 2020	[59]	Phase 3 trial	Gram (−) HAP	0/148 (0)		
Bassetti, 2020	[60]	Phase 3 trial	CR Gram (−) infections	0/101 (0)		
Bradley, 2025	[61]	Phase 2 trial	Pediatric infections	2/53 (3.7)	Defined as renal impairment in 2/29 (6.9%) patients receiving multiple doses of cefiderocol.	
Katsube, 2017	[62]	Prospective cohort study	Gram (−) infections in patients with baseline renal impairment	0/38 (0)		
Falcone, 2022	[63]	Retrospective cohort study	CRAB infections	0/47 (0)		
Campogiani, 2023	[64]	Retrospective cohort study	MDR *A. baumannii* infections	0/11 (0)		
Karruli, 2023	[65]	Retrospective cohort study	MDR Gram (−) infections	0/28 (0)		
Clancy, 2024	[66]	Retrospective cohort study	Gram (−) infections	1/244 (0.4)	Acute interstitial nephritis	
Oliva, 2024	[67]	Retrospective cohort study	CRAB infections	0/50 (0)		
Cipko, 2021	[68]	Case report	*P. aeruginosa* and XDR *A. baumannii* retained spinal hardware infection		Acute interstitial nephritis	
Sollima, 2020	[69]	Case series	MDR Gram (−) CNS infections	0/5 (0)		
*Ceftobiprole*						VigiBase: 6/140 (3%) reports FAERS: 1/94 (1.1%) reports FDA: Reported in ≥2% of patients in clinical trials
Schmitt-Hoffman, 2004	[70]	Phase 1 trial	Healthy subjects	0/16 (0)		
Noel, 2008	[71]	Phase 3 trial	cSSSIs	1/348 (0.3)	Two-fold increase in creatinine upper normal limit in laboratory tests.	
Noel, 2008	[72]	Phase 3 trial	cSSSIs	10/543 (1.9)	Renal related adverse events, not explicitly specified.	
Awad, 2014	[73]	Phase 3 trial	HAP	0/386 (0)		
Bosheva, 2021	[74]	Phase 3 trial	Pneumonia in pediatric patients	0/94 (0)		
Li, 2021	[75]	Phase 1 trial	Healthy subjects	0/12 (0)		
Overcash, 2021	[76]	Phase 3 trial	ABSSIs	0/334 (0)		
Holland, 2023	[77]	Phase 3 trial	*S. aureus* bacteremia	0/191 (0)		
Crapis, 2021	[78]	Retrospective cohort study	Severe CAP or HAP	0/48 (0)		
Arnés García, 2023	[79]	Retrospective cohort study	Various infections	0/227 (0)		
Durante-Mangoni, 2020	[80]	Retrospective cohort study	Various infections	0/29 (0)		
Zampino, 2023	[81]	Retrospective cohort study	Various infections	0/63		
Membrillo De Novales, 2025	[82]	Retrospective cohort study	Various infections	High dose: 9/46 [19.6%, 2/46 (4.3%) drug-related]; Recommended dose: 26/350 (7.4%, unspecified)	AKI	
Zhanel, 2021	[83]	Cross-sectional, survey-based registry study	Various infections	0/38 ^b^		
*Contezolid*						VigiBase: No data NMPA: No data
Eckburg, 2017	[84]	Phase 1 trial	Healthy subjects	0/33 (0)		
Wu, 2018	[85]	Phase 1 trial	Healthy subjects	0/84 (0)		
Wu, 2019	[86]	Phase 1 trial	Healthy subjects	0/68 (0)		
Zhao, 2022	[87]	Phase 3 trial	cSSTIs	0/354 (0)		
Yang, 2023	[88]	Phase 1 trial	Healthy subjects	0/55 (0)		
Li, 2023	[89]	Case series	Various infections	0/3 (0)		
*Gepotidacin*						VigiBase: No data FAERS: No data FDA: NL
O′Riordan, 2017	[90]	Phase 2 trial	ABSSSIs	0/122 (0)		
Taylor, 2018	[91]	Phase 2 trial	Uncomplicated urogenital gonorrhea	0/105 (0)		
Hossain, 2020	[92]	Phase 1 trial	Healthy subjects and patients with CKD	0/32		
Overcash, 2020	[93]	Phase 2a trial	Acute uncomplicated cystitis	0/22 (0)		
Tiffany, 2022	[94]	Phase 1 trial	Healthy adults and elderly subjects	Repeated ascending oral doses in subjects aged 18–60 years: 48/72 (66.7) Repeated dosing in subjects aged ≥ 65 years: 4/29 (13.8)	Proteinuria	
Barth, 2023	[95]	Phase 1 trial	Healthy subjects	0/36 (0)		
Wagenlehner, 2024	[96]	Phase 3 trial	Uncomplicated UTIs	0/1580 (0)		
Ross, 2025	[97]	Phase 3 trial	Uncomplicated urogenital gonorrhea	0/309 (0)		
*Imipenem/cilastatin/relebactam*						VigiBase: 5/63 (4%) reports FAERS: 1 tubulointerstitial nephritis and 1 blood creatinine increase out of 82 reports EudraVigilance: 4/56 (7.1%) reports EMA: listed as uncommon (blood creatinine increase) and rare (acute renal failure, oliguria/anuria, polyuria, urine discoloration) FDA: reported in <1% of patients in a clinical trial
Sims, 2017	[98]	Phase 2 trial	cUTIs	1/99 (1)	Proteinuria	
Rhee, 2018	[99]	Phase 1 trial	Healthy subjects	0/90 (0)		
Bhagunde, 2020	[100]	Phase 1 trial	CKD patients and healthy subjects	0/63 (0)		
Motsch, 2020	[101]	Phase 3 trial	IR bacterial infections	3/29 (10.3)	AKI	
Titov, 2020	[102]	Phase 3 trial	HAP, VAP	0/266 (0)		
Roberts, 2023	[103]	Phase 3 trial	HAP, VAP in patients with CKD	0/266 (0)		
Caniff, 2025	[104]	Retrospective cohort study	Gram (−) infections	1/106 (0.9)	AKI	
Machuca, 2024	[105]	Case series	DTT, *P. aeruginosa* infections	0/14 (0)		
*Lascufloxacin*						VigiBase: 6/63 (8%) reports PMDA: No data
Totsuka, 2019	[106]	Phase 1 trial	Healthy subjects	2/77 (2.6)	Isolated serum creatinine increased	
Takazono, 2024	[107]	Phase 3 trial	Nursing home- and healthcare-associated pneumonia	0/71 (0)		
Iwanaga, 2025	[108]	Phase 3 trial	CAP	1/114 (0.9)	AKI	
Shimada, 2024	[109]	Retrospective cohort study	LRTIs	0/55 (0)		
*Lefamulin*						VigiBase: NR FAERS: NR EudraVigilance: NR EMA: NL FDA: NL
Prince, 2013	[110]	Phase 2 trial	ABSSSIs	0/207 (0)		
Alexander, 2019	[111]	Phase 3 trial	CAP	0/368 (0)		
File, 2019	[112]	Phase 3 trial	CAP	0/273 (0)		
Wicha, 2019	[113]	Phase 1 and 2 trials	Healthy subjects and ABSSSIs	0/207 (0)		
Wicha, 2021	[114]	Phase 1 trial	Subjects with CKD	0/23 (0)		
Hu, 2023	[115]	Phase 1 trial	Healthy subjects	2/20 (5)	AKI	
*Levonadifloxacin*						VigiBase: NR CDSCO: NL
Mehta, 2022	[116]	Retrospective cohort study	ABSSSIs	0/227 (0)		
Mehta, 2022	[117]	Retrospective	Various bacterial infections	0/1229 (0)		
Saseedharan, 2024	[118]	Prospective	Various bacterial infections	0/1266 (0)		
Telkhade, 2024	[119]	Prospective	CAP	0/92 (0)		
*Plazomicin*						VigiBase: 3/11 (15%) reports FAERS: 5 blood creatinine increase and 2 AKI out of 11 reports FDA: Nephrotoxicity warning
Cass, 2011	[120]	Phase 1 trial	Healthy subjects	0/32 (0)		
Connoly, 2018	[121]	Phase 2 trial	cUTIs and acute pyelonephritis	7/96 (2.1)	One patient with AKI, one with mild azotemia, and five with a 0.5 mg/dL increase in creatinine from baseline.	
Wagenlehner, 2019	[122]	Phase 3 trial	cUTIs	32/303 (10.5)	11/303 (3.6%) patients with deterioration of renal function, 21/303 (6.9%) patients had an increase in serum creatinine ≥ 0.5 mg/dL.	
Gall, 2019	[123]	Phase 1 trial	Healthy subjects	0/111 (0)		
*Sulbactam/durlobactam*						VigiBase: NR FAERS: NR FDA: reported in 6% of patients in a clinical trial
O’Donnell, 2019	[124]	Phase 1 trial	Patients with CKD and healthy controls	0/34 (0)		
Kaye, 2023	[125]	Phase 3 trial	*A. baumannii* infections	12/91 (13.2)	AKI	

**Abbreviations**: ABSSSIs: acute bacterial skin and skin structure infections, AKI: acute kidney injury, CAP: community-acquired pneumonia, CDSCO: Central Drugs Standard Control Organization, CKD: chronic kidney disease, CR: carbapenem-resistant, CRAB: carbapenem-resistant *A. baumannii*, DTT: difficult-to-treat, EMA: European Medicines Agency, FAERS: FDA Adverse Event Reporting System, FDA: Food and Drug Administration, HAP: hospital-acquired pneumonia, cIAIs: complicated intra-abdominal infections, IR: imipenem-resistant, LRTI: lower respiratory tract infection, MDR: multidrug resistant, NMPA: National Medical Products Administration, NK: not known, NL: not listed, NR: not reported, PMDA: Pharmaceuticals and Medical Devices Agency, cSSSIs: complicated skin and skin structure infections, cSSTIs: complicated skin and soft tissue infections, XDR: extensively drug-resistant, cUTIs: complicated urinary tract infections. * The studies are grouped by antibiotic and by type, and within each group, they are presented in chronological order from oldest to most recent. ^a^ Results of the ASSEMBLE study have been posted, but they have not been published in a journal at the time of writing. ^b^ In this study, a case of interstitial nephritis was reported, but it was not attributed to ceftobiprole.

## Data Availability

The data used in this study are available upon request.

## Supplementary Materials

The following supporting information can be downloaded at: https://www.mdpi.com/article/10.3390/toxics13070606/s1, Supplementary File S1. Search strategy; Supplementary File S2. PRISMA 2020 Checklist; Supplementary File S3. PRISMA 2020 for Abstracts Checklist; Table S1: Excluded studies after full-text screening; Table S2: Risk of bias assessment for clinical trials with the Jadad scale; Table S3: Risk of bias assessment for observational studies with the Newcastle–Ottawa scale; Table S4: Risk of bias assessment for non-randomized clinical trials (ROBINS-I V2); Table S5: Studies not assessed for risk of bias.

## Abbreviations

ADR	Adverse drug reaction
AKI	Acute kidney injury
CDSCO	Central Drugs Standard Control Organization
CKD	Chronic kidney disease
EMA	European Medicines Agency
FAERS	FDA Adverse Event Reporting System
FDA	Food and Drug Administration
KDIGO	Kidney disease|improving global outcomes
NMPA	National Medical Products Administration
NSAIDs	Non-steroidal anti-inflammatory drugs
PMDA	Pharmaceuticals and Medical Devices Agency
PRISMA	Preferred Reporting Items for Systematic Reviews and Meta-Analyses
RCTs	Randomized controlled trials
