# Supplementary material for: Nephrotoxicity of New Antibiotics: A Systematic Review
Source: Toxics. 2025 Jul 19;13(7):606. doi: 10.3390/toxics13070606 (PMC12299473; doi:10.3390/toxics13070606)
Supplement: Supplementary file 1 [file toxics-13-00606-s001.zip › Supplementary file 1 - Search strategy-7-17.pdf]

Supplementary file 1. Search strategy

Aztreonam/avibactam

PubMed (n=14): (aztreonam-avibactam[tiab] OR aztreonam avibactam[tiab] OR ATM-AVI[tiab] OR Emblaveo[tiab] OR aztreonam-avibactam[Mesh]) AND (nephrotoxicity[tiab] OR nephrotoxic[tiab] OR "kidney injury"[tiab] OR "renal injury"[tiab] OR "renal dysfunction"[tiab] OR "kidney dysfunction"[tiab] OR "renal impairment"[tiab] OR "kidney impairment"[tiab] OR "renal failure"[tiab] OR "kidney failure"[tiab] OR "renal adverse effects"[tiab] OR "kidney adverse effects"[tiab] OR "renal side effects"[tiab] OR "kidney side effects"[tiab] OR "renal toxicity"[tiab] OR "kidney toxicity"[tiab] OR "acute kidney injury"[tiab] OR AKI[tiab] OR "glomerular toxicity"[tiab] OR "tubular toxicity"[tiab] OR "tubular damage"[tiab] OR "glomerular damage"[tiab] OR "renal damage"[tiab] OR "kidney damage"[tiab]) OR ((aztreonam-avibactam[tiab] OR aztreonam avibactam[tiab] OR ATM-AVI[tiab] OR Emblaveo[tiab] OR aztreonam-avibactam[Mesh]) AND (toxicity[tiab] OR "drug toxicity"[tiab] OR "drug safety"[tiab] OR "adverse effects"[tiab] OR "adverse events"[tiab] OR "preclinical toxicity"[tiab] OR "renal safety"[tiab] OR "organ toxicity"[tiab]))

Scopus (n=27): (TITLE-ABS-KEY("aztreonam-avibactam" OR "aztreonam avibactam" OR "ATM-AVI" OR "Emblaveo")) AND (TITLE-ABS-KEY("nephrotoxicity" OR "nephrotoxic" OR "kidney injury" OR "renal injury" OR "renal dysfunction" OR "kidney dysfunction" OR "renal impairment" OR "kidney impairment" OR "renal failure" OR "kidney failure" OR "renal adverse effects" OR "kidney adverse effects" OR "renal side effects" OR "kidney side effects" OR "renal toxicity" OR "kidney toxicity" OR "acute kidney injury" OR "AKI" OR "glomerular toxicity" OR "tubular toxicity" OR "tubular damage" OR "glomerular damage" OR "renal damage" OR "kidney damage")) OR ((TITLE-ABS-KEY("aztreonam-avibactam" OR "aztreonam avibactam" OR "ATM-AVI" OR "Emblaveo")) AND TITLE-ABS-KEY("toxicity" OR "drug toxicity" OR "drug safety" OR "adverse effects" OR "adverse events" OR "preclinical toxicity" OR "renal safety" OR "organ toxicity"))

Web of Science (n=26): (TS=("aztreonam-avibactam" OR "aztreonam avibactam" OR "ATM-AVI" OR "Emblaveo") AND TS=("nephrotoxicity" OR "nephrotoxic" OR "kidney injury" OR "renal injury" OR "renal dysfunction" OR "kidney dysfunction" OR "renal impairment" OR "kidney impairment" OR "renal failure" OR "kidney failure" OR "renal adverse effects" OR "kidney adverse effects" OR "renal side effects" OR "kidney side effects" OR "renal toxicity" OR "kidney toxicity" OR "acute kidney injury" OR "AKI" OR "glomerular toxicity" OR "tubular toxicity" OR "tubular damage" OR "glomerular damage" OR "renal damage" OR "kidney damage")) OR (TS=("aztreonam-avibactam" OR "aztreonam avibactam" OR "ATM-AVI" OR "Emblaveo") AND TS=("toxicity" OR "drug toxicity" OR "drug safety" OR

"adverse effects" OR "adverse events" OR "preclinical toxicity" OR "renal safety" OR "organ toxicity"))

Cefepime/enmetazobactam

PubMed (n=6): (cefepime-enmetazobactam[tiab] OR cefepime enmetazobactam[tiab] OR Exblifep[tiab] OR AAI-101[tiab] OR AAI101[tiab] OR FEP/AAI101[tiab] OR EMT/FEP[tiab] OR cefepime-enmetazobactam[Mesh]) AND (nephrotoxicity[tiab] OR nephrotoxic[tiab] OR "kidney injury"[tiab] OR "renal injury"[tiab] OR "renal dysfunction"[tiab] OR "kidney dysfunction"[tiab] OR "renal impairment"[tiab] OR "kidney impairment"[tiab] OR "renal failure"[tiab] OR "kidney failure"[tiab] OR "renal adverse effects"[tiab] OR "kidney adverse effects"[tiab] OR "renal side effects"[tiab] OR "kidney side effects"[tiab] OR "renal toxicity"[tiab] OR "kidney toxicity"[tiab] OR "acute kidney injury"[tiab] OR AKI[tiab] OR "glomerular toxicity"[tiab] OR "tubular toxicity"[tiab] OR "tubular damage"[tiab] OR "glomerular damage"[tiab] OR "renal damage"[tiab] OR "kidney damage"[tiab]) OR ((cefepime-enmetazobactam[tiab] OR cefepime enmetazobactam[tiab] OR Exblifep[tiab] OR AAI-101[tiab] OR AAI101[tiab] OR FEP/AAI101[tiab] OR EMT/FEP[tiab] OR cefepime-enmetazobactam[Mesh]) AND (toxicity[tiab] OR "drug toxicity"[tiab] OR "drug safety"[tiab] OR "adverse effects"[tiab] OR "adverse events"[tiab] OR "preclinical toxicity"[tiab] OR "renal safety"[tiab] OR "organ toxicity"[tiab]))

Scopus (n=12): (TITLE-ABS-KEY("cefepime-enmetazobactam" OR "cefepime enmetazobactam" OR "Exblifep" OR "AAI-101" OR "AAI101" OR "FEP/AAI101" OR "EMT/FEP")) AND (TITLE-ABS-KEY("nephrotoxicity" OR "nephrotoxic" OR "kidney injury" OR "renal injury" OR "renal dysfunction" OR "kidney dysfunction" OR "renal impairment" OR "kidney impairment" OR "renal failure" OR "kidney failure" OR "renal adverse effects" OR "kidney adverse effects" OR "renal side effects" OR "kidney side effects" OR "renal toxicity" OR "kidney toxicity" OR "acute kidney injury" OR "AKI" OR "glomerular toxicity" OR "tubular toxicity" OR "tubular damage" OR "glomerular damage" OR "renal damage" OR "kidney damage")) OR ((TITLE-ABS-KEY("cefepime-enmetazobactam" OR "cefepime enmetazobactam" OR "Exblifep" OR "AAI-101" OR "AAI101" OR "FEP/AAI101" OR "EMT/FEP")) AND TITLE-ABS-KEY("toxicity" OR "drug toxicity" OR "drug safety" OR "adverse effects" OR "adverse events" OR "preclinical toxicity" OR "renal safety" OR "organ toxicity"))

Web of Science (n=11): (TS=("cefepime-enmetazobactam" OR "cefepime enmetazobactam" OR "Exblifep" OR "AAI-101" OR "AAI101" OR "FEP/AAI101" OR "EMT/FEP") AND TS=("nephrotoxicity" OR "nephrotoxic" OR "kidney injury" OR "renal injury" OR "renal dysfunction" OR "kidney dysfunction" OR "renal impairment" OR "kidney impairment" OR "renal failure" OR "kidney failure" OR "renal adverse effects" OR "kidney adverse effects" OR "renal side effects" OR "kidney side effects" OR "renal toxicity" OR "kidney toxicity" OR "acute kidney injury" OR "AKI" OR "glomerular toxicity" OR "tubular toxicity" OR "tubular

damage" OR "glomerular damage" OR "renal damage" OR "kidney damage")) OR (TS=("cefepime-enmetazobactam" OR "cefepime enmetazobactam" OR "Exblifep" OR "AAI-101" OR "AAI101" OR "FEP/AAI101" OR "EMT/FEP") AND TS=("toxicity" OR "drug toxicity" OR "drug safety" OR "adverse effects" OR "adverse events" OR "preclinical toxicity" OR "renal safety" OR "organ toxicity"))

Cefiderocol

PubMed (n=97): (cefiderocol[tiab] OR S-649266[tiab] OR RSC-649266[tiab] OR Fetroja[tiab] OR Fetcroja[tiab] OR cefiderocol[Mesh]) AND (nephrotoxicity[tiab] OR nephrotoxic[tiab] OR "kidney injury"[tiab] OR "renal injury"[tiab] OR "renal dysfunction"[tiab] OR "kidney dysfunction"[tiab] OR "renal impairment"[tiab] OR "kidney impairment"[tiab] OR "renal failure"[tiab] OR "kidney failure"[tiab] OR "renal adverse effects"[tiab] OR "kidney adverse effects"[tiab] OR "renal side effects"[tiab] OR "kidney side effects"[tiab] OR "renal toxicity"[tiab] OR "kidney toxicity"[tiab] OR "acute kidney injury"[tiab] OR AKI[tiab] OR "glomerular toxicity"[tiab] OR "tubular toxicity"[tiab] OR "tubular damage"[tiab] OR "glomerular damage"[tiab] OR "renal damage"[tiab] OR "kidney damage"[tiab]) OR ((cefiderocol[tiab] OR S-649266[tiab] OR RSC-649266[tiab] OR Fetroja[tiab] OR Fetcroja[tiab] OR cefiderocol[Mesh]) AND (toxicity[tiab] OR "drug toxicity"[tiab] OR "drug safety"[tiab] OR "adverse effects"[tiab] OR "adverse events"[tiab] OR "preclinical toxicity"[tiab] OR "renal safety"[tiab] OR "organ toxicity"[tiab]))

Scopus (n=328) (TITLE-ABS-KEY("cefiderocol" OR "S-649266" OR "RSC-649266" OR "Fetroja" OR "Fetcroja")) AND (TITLE-ABS-KEY("nephrotoxicity" OR "nephrotoxic" OR "kidney injury" OR "renal injury" OR "renal dysfunction" OR "kidney dysfunction" OR "renal impairment" OR "kidney impairment" OR "renal failure" OR "kidney failure" OR "renal adverse effects" OR "kidney adverse effects" OR "renal side effects" OR "kidney side effects" OR "renal toxicity" OR "kidney toxicity" OR "acute kidney injury" OR "AKI" OR "glomerular toxicity" OR "tubular toxicity" OR "tubular damage" OR "glomerular damage" OR "renal damage" OR "kidney damage")) OR ((TITLE-ABS-KEY("cefiderocol" OR "S-649266" OR "RSC-649266" OR "Fetroja" OR "Fetcroja")) AND TITLE-ABS-KEY("toxicity" OR "drug toxicity" OR "drug safety" OR "adverse effects" OR "adverse events" OR "preclinical toxicity" OR "renal safety" OR "organ toxicity"))

Web of Science (n=157): (TS=("cefiderocol" OR "S-649266" OR "RSC-649266" OR "Fetroja" OR "Fetcroja")AND TS=("nephrotoxicity" OR "nephrotoxic" OR "kidney injury" OR "renal injury" OR "renal dysfunction" OR "kidney dysfunction" OR "renal impairment" OR "kidney impairment" OR "renal failure" OR "kidney failure" OR "renal adverse effects" OR "kidney adverse effects" OR "renal side effects" OR "kidney side effects" OR "renal toxicity" OR "kidney toxicity" OR "acute kidney injury" OR "AKI" OR "glomerular toxicity" OR "tubular toxicity" OR "tubular damage" OR "glomerular damage" OR "renal damage" OR "kidney damage")) OR (TS=("cefiderocol" OR "S-649266" OR "RSC-649266" OR "Fetroja" OR "Fetcroja")

AND TS=("toxicity" OR "drug toxicity" OR "drug safety" OR "adverse effects" OR "adverse events" OR "preclinical toxicity" OR "renal safety" OR "organ toxicity"))

Ceftobiprole

PubMed (n=67): (ceftobiprole[tiab] OR BAL27862[tiab] OR ceftobiprole[Mesh]) AND (nephrotoxicity[tiab] OR nephrotoxic[tiab] OR "kidney injury"[tiab] OR "renal injury"[tiab] OR "renal dysfunction"[tiab] OR "kidney dysfunction"[tiab] OR "renal impairment"[tiab] OR "kidney impairment"[tiab] OR "renal failure"[tiab] OR "kidney failure"[tiab] OR "renal adverse effects"[tiab] OR "kidney adverse effects"[tiab] OR "renal side effects"[tiab] OR "kidney side effects"[tiab] OR "renal toxicity"[tiab] OR "kidney toxicity"[tiab] OR "acute kidney injury"[tiab] OR AKI[tiab] OR "glomerular toxicity"[tiab] OR "tubular toxicity"[tiab] OR "tubular damage"[tiab] OR "glomerular damage"[tiab] OR "renal damage"[tiab] OR "kidney damage"[tiab]) OR ((ceftobiprole[tiab] OR BAL27862[tiab] OR ceftobiprole[Mesh]) AND (toxicity[tiab] OR "drug toxicity"[tiab] OR "drug safety"[tiab] OR "adverse effects"[tiab] OR "adverse events"[tiab] OR "preclinical toxicity"[tiab] OR "renal safety"[tiab] OR "organ toxicity"[tiab]))

Scopus (n=366): (TITLE-ABS-KEY ("ceftobiprole" OR "BAL27862")) AND (TITLE-ABS-KEY ("nephrotoxicity" OR "nephrotoxic" OR "kidney injury" OR "renal injury" OR "renal dysfunction" OR "kidney dysfunction" OR "renal impairment" OR "kidney impairment" OR "renal failure" OR "kidney failure" OR "renal adverse effects" OR "kidney adverse effects" OR "renal side effects" OR "kidney side effects" OR "renal toxicity" OR "kidney toxicity" OR "acute kidney injury" OR "AKI" OR "glomerular toxicity" OR "tubular toxicity" OR "tubular damage" OR "glomerular damage" OR "renal damage" OR "kidney damage")) OR ((TITLE-ABS-KEY ("ceftobiprole" OR "BAL27862")) AND TITLE-ABS-KEY ("toxicity" OR "drug toxicity" OR "drug safety" OR "adverse effects" OR "adverse events" OR "preclinical toxicity" OR "renal safety" OR "organ toxicity"))

Web of Science (n=132): (TS=("ceftobiprole" OR "BAL27862")AND TS=("nephrotoxicity" OR "nephrotoxic" OR "kidney injury" OR "renal injury" OR "renal dysfunction" OR "kidney dysfunction" OR "renal impairment" OR "kidney impairment" OR "renal failure" OR "kidney failure" OR "renal adverse effects" OR "kidney adverse effects" OR "renal side effects" OR "kidney side effects" OR "renal toxicity" OR "kidney toxicity" OR "acute kidney injury" OR "AKI" OR "glomerular toxicity" OR "tubular toxicity" OR "tubular damage" OR "glomerular damage" OR "renal damage" OR "kidney damage")) OR (TS=("ceftobiprole" OR "BAL27862") AND TS=("toxicity" OR "drug toxicity" OR "drug safety" OR "adverse effects" OR "adverse events" OR "preclinical toxicity" OR "renal safety" OR "organ toxicity"))

Contezolid

PubMed (n=27): (contezolid[tiab] OR MRX-I[tiab] OR contezolid[Mesh]) AND (nephrotoxicity[tiab] OR nephrotoxic[tiab] OR "kidney injury"[tiab] OR "renal

injury"[tiab] OR "renal dysfunction"[tiab] OR "kidney dysfunction"[tiab] OR "renal impairment"[tiab] OR "kidney impairment"[tiab] OR "renal failure"[tiab] OR "kidney failure"[tiab] OR "renal adverse effects"[tiab] OR "kidney adverse effects"[tiab] OR "renal side effects"[tiab] OR "kidney side effects"[tiab] OR "renal toxicity"[tiab] OR "kidney toxicity"[tiab] OR "acute kidney injury"[tiab] OR AKI[tiab] OR "glomerular toxicity"[tiab] OR "tubular toxicity"[tiab] OR "tubular damage"[tiab] OR "glomerular damage"[tiab] OR "renal damage"[tiab] OR "kidney damage"[tiab]) OR ((contezolid[tiab] OR MRX-I[tiab] OR contezolid[Mesh]) AND (toxicity[tiab] OR "drug toxicity"[tiab] OR "drug safety"[tiab] OR "adverse effects"[tiab] OR "adverse events"[tiab] OR "preclinical toxicity"[tiab] OR "renal safety"[tiab] OR "organ toxicity"[tiab]))

Scopus (n=58): (TITLE-ABS-KEY("contezolid" OR "MRX-I")) AND (TITLE-ABS-KEY("nephrotoxicity" OR "nephrotoxic" OR "kidney injury" OR "renal injury" OR "renal dysfunction" OR "kidney dysfunction" OR "renal impairment" OR "kidney impairment" OR "renal failure" OR "kidney failure" OR "renal adverse effects" OR "kidney adverse effects" OR "renal side effects" OR "kidney side effects" OR "renal toxicity" OR "kidney toxicity" OR "acute kidney injury" OR "AKI" OR "glomerular toxicity" OR "tubular toxicity" OR "tubular damage" OR "glomerular damage" OR "renal damage" OR "kidney damage")) OR ((TITLE-ABS-KEY("contezolid" OR "MRX-I")) AND TITLE-ABS-KEY("toxicity" OR "drug toxicity" OR "drug safety" OR "adverse effects" OR "adverse events" OR "preclinical toxicity" OR "renal safety" OR "organ toxicity"))

Web of Science (n=43): (TS=("contezolid" OR "MRX-I")AND TS=("nephrotoxicity" OR "nephrotoxic" OR "kidney injury" OR "renal injury" OR "renal dysfunction" OR "kidney dysfunction" OR "renal impairment" OR "kidney impairment" OR "renal failure" OR "kidney failure" OR "renal adverse effects" OR "kidney adverse effects" OR "renal side effects" OR "kidney side effects" OR "renal toxicity" OR "kidney toxicity" OR "acute kidney injury" OR "AKI" OR "glomerular toxicity" OR "tubular toxicity" OR "tubular damage" OR "glomerular damage" OR "renal damage" OR "kidney damage")) OR (TS=("contezolid" OR "MRX-I")AND TS=("toxicity" OR "drug toxicity" OR "drug safety" OR "adverse effects" OR "adverse events" OR "preclinical toxicity" OR "renal safety" OR "organ toxicity"))

Gepotidacin

PubMed (n=9): (gepotidacin[tiab] OR GSK2140944[tiab] OR gepotidacin[Mesh]) AND (nephrotoxicity[tiab] OR nephrotoxic[tiab] OR "kidney injury"[tiab] OR "renal injury"[tiab] OR "renal dysfunction"[tiab] OR "kidney dysfunction"[tiab] OR "renal impairment"[tiab] OR "kidney impairment"[tiab] OR "renal failure"[tiab] OR "kidney failure"[tiab] OR "renal adverse effects"[tiab] OR "kidney adverse effects"[tiab] OR "renal side effects"[tiab] OR "kidney side effects"[tiab] OR "renal toxicity"[tiab] OR "kidney toxicity"[tiab] OR "acute kidney injury"[tiab] OR AKI[tiab] OR "glomerular toxicity"[tiab] OR "tubular toxicity"[tiab] OR "tubular damage"[tiab] OR "glomerular

damage"[tiab] OR "renal damage"[tiab] OR "kidney damage"[tiab]) OR ((gepotidacin[tiab] OR GSK2140944[tiab] OR gepotidacin[Mesh]) AND (toxicity[tiab] OR "drug toxicity"[tiab] OR "drug safety"[tiab] OR "adverse effects"[tiab] OR "adverse events"[tiab] OR "preclinical toxicity"[tiab] OR "renal safety"[tiab] OR "organ toxicity"[tiab]))

Scopus (n=47): (TITLE-ABS-KEY("gepotidacin" OR "GSK2140944")) AND (TITLE-ABS-KEY("nephrotoxicity" OR "nephrotoxic" OR "kidney injury" OR "renal injury" OR "renal dysfunction" OR "kidney dysfunction" OR "renal impairment" OR "kidney impairment" OR "renal failure" OR "kidney failure" OR "renal adverse effects" OR "kidney adverse effects" OR "renal side effects" OR "kidney side effects" OR "renal toxicity" OR "kidney toxicity" OR "acute kidney injury" OR "AKI" OR "glomerular toxicity" OR "tubular toxicity" OR "tubular damage" OR "glomerular damage" OR "renal damage" OR "kidney damage")) OR ((TITLE-ABS-KEY("gepotidacin" OR "GSK2140944"))AND TITLE-ABS-KEY("toxicity" OR "drug toxicity" OR "drug safety" OR "adverse effects" OR "adverse events" OR "preclinical toxicity" OR "renal safety" OR "organ toxicity"))

Web of Science (n=15): (TS=("gepotidacin" OR "GSK2140944")AND TS=("nephrotoxicity" OR "nephrotoxic" OR "kidney injury" OR "renal injury" OR "renal dysfunction" OR "kidney dysfunction" OR "renal impairment" OR "kidney impairment" OR "renal failure" OR "kidney failure" OR "renal adverse effects" OR "kidney adverse effects" OR "renal side effects" OR "kidney side effects" OR "renal toxicity" OR "kidney toxicity" OR "acute kidney injury" OR "AKI" OR "glomerular toxicity" OR "tubular toxicity" OR "tubular damage" OR "glomerular damage" OR "renal damage" OR "kidney damage"))OR(TS=("gepotidacin" OR "GSK2140944")AND TS=("toxicity" OR "drug toxicity" OR "drug safety" OR "adverse effects" OR "adverse events" OR "preclinical toxicity" OR "renal safety" OR "organ toxicity"))

Imipenem/cilastatin/relebactam

Pubmed (n= 29): (imipenem-cilastatin-relebactam[tiab] OR imipenem cilastatin relebactam[tiab] OR recarbrio[tiab] OR sulbactam-durlobactam[Mesh]) AND (nephrotoxicity[tiab] OR nephrotoxic[tiab] OR "kidney injury"[tiab] OR "renal injury"[tiab] OR "renal dysfunction"[tiab] OR "kidney dysfunction"[tiab] OR "renal impairment"[tiab] OR "kidney impairment"[tiab] OR "renal failure"[tiab] OR "kidney failure"[tiab] OR "renal adverse effects"[tiab] OR "kidney adverse effects"[tiab] OR "renal side effects"[tiab] OR "kidney side effects"[tiab] OR "renal toxicity"[tiab] OR "kidney toxicity"[tiab] OR "acute kidney injury"[tiab] OR AKI[tiab] OR "glomerular toxicity"[tiab] OR "tubular toxicity"[tiab] OR "tubular damage"[tiab] OR "glomerular damage"[tiab] OR "renal damage"[tiab] OR "kidney damage"[tiab]) OR ((imipenem-cilastatin-relebactam[tiab] OR imipenem cilastatin relebactam[tiab] OR Recarbrio[tiab] OR imipenem-cilastatin-relebactam[Mesh]) AND (toxicity[tiab] OR "drug toxicity"[tiab] OR "drug safety"[tiab] OR "adverse effects"[tiab] OR "adverse

events"[tiab] OR "preclinical toxicity"[tiab] OR "renal safety"[tiab] OR "organ toxicity"[tiab]))

Scopus (n=56): TITLE-ABS-KEY ("imipenem-cilastatin-relebactam" OR "imipenem cilastatin relebactam" OR "Recarbrio") AND (TITLE-ABS-KEY ("nephrotoxicity" OR "nephrotoxic" OR "kidney injury" OR "renal injury" OR "renal dysfunction" OR "kidney dysfunction" OR "renal impairment" OR "kidney impairment" OR "renal failure" OR "kidney failure" OR "renal adverse effects" OR "kidney adverse effects" OR "renal side effects" OR "kidney side effects" OR "renal toxicity" OR "kidney toxicity" OR "acute kidney injury" OR "AKI" OR "glomerular toxicity" OR "tubular toxicity" OR "tubular damage" OR "glomerular damage" OR "renal damage" OR "kidney damage") OR TITLE-ABS-KEY ("toxicity" OR "drug toxicity" OR "drug safety" OR "adverse effects" OR "adverse events" OR "preclinical toxicity" OR "renal safety" OR "organ toxicity"))

Web of science (n=28): TS=(("imipenem-cilastatin-relebactam" OR "imipenem cilastatin relebactam" OR "Recarbrio") AND ("nephrotoxicity" OR "nephrotoxic" OR "kidney injury" OR "renal injury" OR "renal dysfunction" OR "kidney dysfunction" OR "renal impairment" OR "kidney impairment" OR "renal failure" OR "kidney failure" OR "renal adverse effects" OR "kidney adverse effects" OR "renal side effects" OR "kidney side effects" OR "renal toxicity" OR "kidney toxicity" OR "acute kidney injury" OR "AKI" OR "glomerular toxicity" OR "tubular toxicity" OR "tubular damage" OR "glomerular damage" OR "renal damage" OR "kidney damage")) OR TS=(("imipenem-cilastatin-relebactam" OR "imipenem cilastatin relebactam" OR "Recarbrio") AND ("toxicity" OR "drug toxicity" OR "drug safety" OR "adverse effects" OR "adverse events" OR "preclinical toxicity" OR "renal safety" OR "organ toxicity"))

Lascufloxacin

PubMed (n=6): (lascufloxacin[tiab] OR KRP-AM1977[tiab] OR KRP-AM1977X[tiab] OR KRP-AM1977Y[tiab] OR lascufloxacin[Mesh]) AND (nephrotoxicity[tiab] OR nephrotoxic[tiab] OR "kidney injury"[tiab] OR "renal injury"[tiab] OR "renal dysfunction"[tiab] OR "kidney dysfunction"[tiab] OR "renal impairment"[tiab] OR "kidney impairment"[tiab] OR "renal failure"[tiab] OR "kidney failure"[tiab] OR "renal adverse effects"[tiab] OR "kidney adverse effects"[tiab] OR "renal side effects"[tiab] OR "kidney side effects"[tiab] OR "renal toxicity"[tiab] OR "kidney toxicity"[tiab] OR "acute kidney injury"[tiab] OR AKI[tiab] OR "glomerular toxicity"[tiab] OR "tubular toxicity"[tiab] OR "tubular damage"[tiab] OR "glomerular damage"[tiab] OR "renal damage"[tiab] OR "kidney damage"[tiab]) OR ((lascufloxacin[tiab] OR KRP-AM1977[tiab] OR KRP-AM1977X[tiab] OR KRP-AM1977Y[tiab] OR lascufloxacin[Mesh]) AND (toxicity[tiab] OR "drug toxicity"[tiab] OR "drug safety"[tiab] OR "adverse effects"[tiab] OR "adverse

events"[tiab] OR "preclinical toxicity"[tiab] OR "renal safety"[tiab] OR "organ toxicity"[tiab]))

Scopus (n=22): (TITLE-ABS-KEY(lascufloxacin OR "KRP-AM1977" OR "KRP-AM1977X" OR "KRP-AM1977Y")) AND (TITLE-ABS-KEY(nephrotoxicity OR nephrotoxic OR "kidney injury" OR "renal injury" OR "renal dysfunction" OR "kidney dysfunction" OR "renal impairment" OR "kidney impairment" OR "renal failure" OR "kidney failure" OR "renal adverse effects" OR "kidney adverse effects" OR "renal side effects" OR "kidney side effects" OR "renal toxicity" OR "kidney toxicity" OR "acute kidney injury" OR AKI OR "glomerular toxicity" OR "tubular toxicity" OR "tubular damage" OR "glomerular damage" OR "renal damage" OR "kidney damage")) OR ((TITLE-ABS-KEY(lascufloxacin OR "KRP-AM1977" OR "KRP-AM1977X" OR "KRP-AM1977Y")) AND (TITLE-ABS-KEY(toxicity OR "drug toxicity" OR "drug safety" OR "adverse effects" OR "adverse events" OR "preclinical toxicity" OR "renal safety" OR "organ toxicity")))

Web of Science (n=10): (TS=("lascufloxacin" OR "KRP-AM1977" OR "KRP-AM1977X" OR "KRP-AM1977Y")AND TS=("nephrotoxicity" OR "nephrotoxic" OR "kidney injury" OR "renal injury" OR "renal dysfunction" OR "kidney dysfunction" OR "renal impairment" OR "kidney impairment" OR "renal failure" OR "kidney failure" OR "renal adverse effects" OR "kidney adverse effects" OR "renal side effects" OR "kidney side effects" OR "renal toxicity" OR "kidney toxicity" OR "acute kidney injury" OR "AKI" OR "glomerular toxicity" OR "tubular toxicity" OR "tubular damage" OR "glomerular damage" OR "renal damage" OR "kidney damage"))OR(TS=("lascufloxacin" OR "KRP-AM1977" OR "KRP-AM1977X" OR "KRP-AM1977Y")AND TS=("toxicity" OR "drug toxicity" OR "drug safety" OR "adverse effects" OR "adverse events" OR "preclinical toxicity" OR "renal safety" OR "organ toxicity"))

Lefamulin

PubMed (n=21): (lefamulin[tiab] OR BC-3781[tiab] OR BC3781[tiab] OR Xenleta[tiab] OR lefamulin[Mesh]) AND (nephrotoxicity[tiab] OR nephrotoxic[tiab] OR "kidney injury"[tiab] OR "renal injury"[tiab] OR "renal dysfunction"[tiab] OR "kidney dysfunction"[tiab] OR "renal impairment"[tiab] OR "kidney impairment"[tiab] OR "renal failure"[tiab] OR "kidney failure"[tiab] OR "renal adverse effects"[tiab] OR "kidney adverse effects"[tiab] OR "renal side effects"[tiab] OR "kidney side effects"[tiab] OR "renal toxicity"[tiab] OR "kidney toxicity"[tiab] OR "acute kidney injury"[tiab] OR AKI[tiab] OR "glomerular toxicity"[tiab] OR "tubular toxicity"[tiab] OR "tubular damage"[tiab] OR "glomerular damage"[tiab] OR "renal damage"[tiab] OR "kidney damage"[tiab]) OR ((lefamulin[tiab] OR BC-3781[tiab] OR BC3781[tiab] OR Xenleta[tiab] OR lefamulin[Mesh]) AND (toxicity[tiab] OR "drug toxicity"[tiab] OR "drug safety"[tiab] OR "adverse effects"[tiab] OR "adverse events"[tiab] OR "preclinical toxicity"[tiab] OR "renal safety"[tiab] OR "organ toxicity"[tiab]))

Scopus (n=81): (TITLE-ABS-KEY(lefamulin OR "BC-3781" OR BC3781 OR Xenleta)) AND (TITLE-ABS-KEY(nephrotoxicity OR nephrotoxic OR "kidney injury" OR "renal injury" OR "renal dysfunction" OR "kidney dysfunction" OR "renal impairment" OR "kidney impairment" OR "renal failure" OR "kidney failure" OR "renal adverse effects" OR "kidney adverse effects" OR "renal side effects" OR "kidney side effects" OR "renal toxicity" OR "kidney toxicity" OR "acute kidney injury" OR AKI OR "glomerular toxicity" OR "tubular toxicity" OR "tubular damage" OR "glomerular damage" OR "renal damage" OR "kidney damage")) OR ((TITLE-ABS-KEY(lefamulin OR "BC-3781" OR BC3781 OR Xenleta)) AND (TITLE-ABS-KEY(toxicity OR "drug toxicity" OR "drug safety" OR "adverse effects" OR "adverse events" OR "preclinical toxicity" OR "renal safety" OR "organ toxicity")))

Web of Science (n=34): (TS=("lefamulin" OR "BC-3781" OR "BC3781" OR "Xenleta")AND TS=("nephrotoxicity" OR "nephrotoxic" OR "kidney injury" OR "renal injury" OR "renal dysfunction" OR "kidney dysfunction" OR "renal impairment" OR "kidney impairment" OR "renal failure" OR "kidney failure" OR "renal adverse effects" OR "kidney adverse effects" OR "renal side effects" OR "kidney side effects" OR "renal toxicity" OR "kidney toxicity" OR "acute kidney injury" OR "AKI" OR "glomerular toxicity" OR "tubular toxicity" OR "tubular damage" OR "glomerular damage" OR "renal damage" OR "kidney damage")) OR (TS=("lefamulin" OR "BC-3781" OR "BC3781" OR "Xenleta")AND TS=("toxicity" OR "drug toxicity" OR "drug safety" OR "adverse effects" OR "adverse events" OR "preclinical toxicity" OR "renal safety" OR "organ toxicity"))

Levonadifloxacin

PubMed (n=9): (levonadifloxacin[tiab] OR WCK 771[tiab] OR alalevonadifloxacin[tiab] OR WCK 2349[tiab] OR levonadifloxacin[Mesh]) AND (nephrotoxicity[tiab] OR nephrotoxic[tiab] OR "kidney injury"[tiab] OR "renal injury"[tiab] OR "renal dysfunction"[tiab] OR "kidney dysfunction"[tiab] OR "renal impairment"[tiab] OR "kidney impairment"[tiab] OR "renal failure"[tiab] OR "kidney failure"[tiab] OR "renal adverse effects"[tiab] OR "kidney adverse effects"[tiab] OR "renal side effects"[tiab] OR "kidney side effects"[tiab] OR "renal toxicity"[tiab] OR "kidney toxicity"[tiab] OR "acute kidney injury"[tiab] OR AKI[tiab] OR "glomerular toxicity"[tiab] OR "tubular toxicity"[tiab] OR "tubular damage"[tiab] OR "glomerular damage"[tiab] OR "renal damage"[tiab] OR "kidney damage"[tiab]) OR ((levonadifloxacin[tiab] OR WCK 771[tiab] OR alalevonadifloxacin[tiab] OR WCK 2349[tiab] OR levonadifloxacin[Mesh]) AND (toxicity[tiab] OR "drug toxicity"[tiab] OR "drug safety"[tiab] OR "adverse effects"[tiab] OR "adverse events"[tiab] OR "preclinical toxicity"[tiab] OR "renal safety"[tiab] OR "organ toxicity"[tiab]))

Scopus (n=28): (TITLE-ABS-KEY(levonadifloxacin OR "WCK 771" OR alalevonadifloxacin OR "WCK 2349")) AND (TITLE-ABS-KEY(nephrotoxicity OR nephrotoxic OR "kidney injury" OR "renal injury" OR "renal dysfunction" OR "kidney dysfunction" OR "renal impairment" OR "kidney impairment" OR "renal

failure" OR "kidney failure" OR "renal adverse effects" OR "kidney adverse effects" OR "renal side effects" OR "kidney side effects" OR "renal toxicity" OR "kidney toxicity" OR "acute kidney injury" OR AKI OR "glomerular toxicity" OR "tubular toxicity" OR "tubular damage" OR "glomerular damage" OR "renal damage" OR "kidney damage")) OR ((TITLE-ABS-KEY(levonadifloxacin OR "WCK 771" OR alalevonadifloxacin OR "WCK 2349")) AND (TITLE-ABS-KEY(toxicity OR "drug toxicity" OR "drug safety" OR "adverse effects" OR "adverse events" OR "preclinical toxicity" OR "renal safety" OR "organ toxicity")))

Web of Science (n=18): (TS=("levonadifloxacin" OR "WCK 771" OR "alalevonadifloxacin" OR "WCK 2349")AND TS=("nephrotoxicity" OR "nephrotoxic" OR "kidney injury" OR "renal injury" OR "renal dysfunction" OR "kidney dysfunction" OR "renal impairment" OR "kidney impairment" OR "renal failure" OR "kidney failure" OR "renal adverse effects" OR "kidney adverse effects" OR "renal side effects" OR "kidney side effects" OR "renal toxicity" OR "kidney toxicity" OR "acute kidney injury" OR "AKI" OR "glomerular toxicity" OR "tubular toxicity" OR "tubular damage" OR "glomerular damage" OR "renal damage" OR "kidney damage")) OR (TS=("levonadifloxacin" OR "WCK 771" OR "alalevonadifloxacin" OR "WCK 2349") AND TS=("toxicity" OR "drug toxicity" OR "drug safety" OR "adverse effects" OR "adverse events" OR "preclinical toxicity" OR "renal safety" OR "organ toxicity"))

Plazomicin

PubMed (n=35): (plazomicin[tiab] OR Zemdri[tiab] OR plazomicin[Mesh]) AND (nephrotoxicity[tiab] OR nephrotoxic[tiab] OR "kidney injury"[tiab] OR "renal injury"[tiab] OR "renal dysfunction"[tiab] OR "kidney dysfunction"[tiab] OR "renal impairment"[tiab] OR "kidney impairment"[tiab] OR "renal failure"[tiab] OR "kidney failure"[tiab] OR "renal adverse effects"[tiab] OR "kidney adverse effects"[tiab] OR "renal side effects"[tiab] OR "kidney side effects"[tiab] OR "renal toxicity"[tiab] OR "kidney toxicity"[tiab] OR "acute kidney injury"[tiab] OR AKI[tiab] OR "glomerular toxicity"[tiab] OR "tubular toxicity"[tiab] OR "tubular damage"[tiab] OR "glomerular damage"[tiab] OR "renal damage"[tiab] OR "kidney damage"[tiab]) OR ((plazomicin[tiab] OR Zemdri[tiab] OR plazomicin[Mesh]) AND (toxicity[tiab] OR "drug toxicity"[tiab] OR "drug safety"[tiab] OR "adverse effects"[tiab] OR "adverse events"[tiab] OR "preclinical toxicity"[tiab] OR "renal safety"[tiab] OR "organ toxicity"[tiab]))

Scopus (n=168):(TITLE-ABS-KEY("plazomicin" OR "Zemdri")) AND (TITLE-ABS-KEY("nephrotoxicity" OR "nephrotoxic" OR "kidney injury" OR "renal injury" OR "renal dysfunction" OR "kidney dysfunction" OR "renal impairment" OR "kidney impairment" OR "renal failure" OR "kidney failure" OR "renal adverse effects" OR "kidney adverse effects" OR "renal side effects" OR "kidney side effects" OR "renal toxicity" OR "kidney toxicity" OR "acute kidney injury" OR "AKI" OR "glomerular toxicity" OR "tubular toxicity" OR "tubular damage" OR "glomerular damage" OR

"renal damage" OR "kidney damage")) OR ((TITLE-ABS-KEY("plazomicin" OR "Zemdri")) AND TITLE-ABS-KEY("toxicity" OR "drug toxicity" OR "drug safety" OR "adverse effects" OR "adverse events" OR "preclinical toxicity" OR "renal safety" OR "organ toxicity"))

Web of Science (n=71): (TS=("plazomicin" OR "Zemdri")) AND (TS=("nephrotoxicity" OR "nephrotoxic" OR "kidney injury" OR "renal injury" OR "renal dysfunction" OR "kidney dysfunction" OR "renal impairment" OR "kidney impairment" OR "renal failure" OR "kidney failure" OR "renal adverse effects" OR "kidney adverse effects" OR "renal side effects" OR "kidney side effects" OR "renal toxicity" OR "kidney toxicity" OR "acute kidney injury" OR "AKI" OR "glomerular toxicity" OR "tubular toxicity" OR "tubular damage" OR "glomerular damage" OR "renal damage" OR "kidney damage")) OR (TS=("plazomicin" OR "Zemdri") AND TS=("toxicity" OR "drug toxicity" OR "drug safety" OR "adverse effects" OR "adverse events" OR "preclinical toxicity" OR "renal safety" OR "organ toxicity"))

Sulbactam/durlobactam

PubMed (n=10): (sulbactam-durlobactam[tiab] OR sulbactam durlobactam[tiab] OR SUL-DUR[tiab] OR Xacduro[tiab] OR sulbactam-durlobactam[Mesh]) AND (nephrotoxicity[tiab] OR nephrotoxic[tiab] OR "kidney injury"[tiab] OR "renal injury"[tiab] OR "renal dysfunction"[tiab] OR "kidney dysfunction"[tiab] OR "renal impairment"[tiab] OR "kidney impairment"[tiab] OR "renal failure"[tiab] OR "kidney failure"[tiab] OR "renal adverse effects"[tiab] OR "kidney adverse effects"[tiab] OR "renal side effects"[tiab] OR "kidney side effects"[tiab] OR "renal toxicity"[tiab] OR "kidney toxicity"[tiab] OR "acute kidney injury"[tiab] OR AKI[tiab] OR "glomerular toxicity"[tiab] OR "tubular toxicity"[tiab] OR "tubular damage"[tiab] OR "glomerular damage"[tiab] OR "renal damage"[tiab] OR "kidney damage"[tiab]) OR ((sulbactam-durlobactam[tiab] OR sulbactam durlobactam[tiab] OR SUL-DUR[tiab] OR Xacduro[tiab] OR sulbactam-durlobactam[Mesh]) AND (toxicity[tiab] OR "drug toxicity"[tiab] OR "drug safety"[tiab] OR "adverse effects"[tiab] OR "adverse events"[tiab] OR "preclinical toxicity"[tiab] OR "renal safety"[tiab] OR "organ toxicity"[tiab]))

Scopus (n=16): (TITLE-ABS-KEY("sulbactam-durlobactam" OR "sulbactam durlobactam" OR "SUL-DUR" OR "Xacduro")) AND (TITLE-ABS-KEY("nephrotoxicity" OR "nephrotoxic" OR "kidney injury" OR "renal injury" OR "renal dysfunction" OR "kidney dysfunction" OR "renal impairment" OR "kidney impairment" OR "renal failure" OR "kidney failure" OR "renal adverse effects" OR "kidney adverse effects" OR "renal side effects" OR "kidney side effects" OR "renal toxicity" OR "kidney toxicity" OR "acute kidney injury" OR "AKI" OR "glomerular toxicity" OR "tubular toxicity" OR "tubular damage" OR "glomerular damage" OR "renal damage" OR "kidney damage"))OR ((TITLE-ABS-KEY("sulbactam-durlobactam" OR "sulbactam durlobactam" OR "SUL-DUR" OR "Xacduro"))AND TITLE-ABS-KEY("toxicity" OR "drug toxicity" OR "drug safety" OR "adverse

effects" OR "adverse events" OR "preclinical toxicity" OR "renal safety" OR "organ toxicity"))

Web of Science (n=20): (TS=("sulbactam-durlobactam" OR "sulbactam durlobactam" OR "SUL-DUR" OR "Xacduro")AND TS=("nephrotoxicity" OR "nephrotoxic" OR "kidney injury" OR "renal injury" OR "renal dysfunction" OR "kidney dysfunction" OR "renal impairment" OR "kidney impairment" OR "renal failure" OR "kidney failure" OR "renal adverse effects" OR "kidney adverse effects" OR "renal side effects" OR "kidney side effects" OR "renal toxicity" OR "kidney toxicity" OR "acute kidney injury" OR "AKI" OR "glomerular toxicity" OR "tubular toxicity" OR "tubular damage" OR "glomerular damage" OR "renal damage" OR "kidney damage")) OR (TS=("sulbactam-durlobactam" OR "sulbactam durlobactam" OR "SUL-DUR" OR "Xacduro")AND TS=("toxicity" OR "drug toxicity" OR "drug safety" OR "adverse effects" OR "adverse events" OR "preclinical toxicity" OR "renal safety" OR "organ toxicity"))
