# Supplementary material for: Nephrotoxicity of New Antibiotics: A Systematic Review
Source: Toxics. 2025 Jul 19;13(7):606. doi: 10.3390/toxics13070606 (PMC12299473; doi:10.3390/toxics13070606)
Supplement: Supplementary file 1 [file toxics-13-00606-s001.zip › Supplementary Table S1 - Excluded studies-7-17.pdf]

Supplementary Table S1. Excluded studies after full-text screening.

Study	Reason for exclusion
10.1007/s40265-024-02035-2	Review
10.2147/IDR.S225553	Study protocol
10.1016/j.jgar.2021.02.004 2213-7165	Review
10.1186/s40360-025-00894-3	Data mining study
10.3390/microorganisms11040984	Combination therapy
10.3390/antibiotics13111048	Review
10.3390/antibiotics13050445	Review
10.1016/j.nwh.2020.07.006	Review
10.6084/ m9.figshare.11792034	Review
10.1080/14787210.2016.1244481	Review
10.1016/j.diagmicrobio.2008.03.004	Review
10.1080/14656566.2018.1516749	Review
10.2217/ fmb-2021-0121	Post-hoc analysis
CMI, 13 (suppl 2), 25–29	Review
Eastern Journal of Medicine 16 (2011) 1-8	Review
10.2217/fmb.15.115	Review
10.2217/fmb-2021-0042	Post-hoc analysis
10.1177/10600280241293773	Review
10.1358/dot.2022.58.7.3389002	Review
10.5582/ddt.2022.01025	Commentary
10.1128/aac.02430-21 1	PopPk analysis
10.1007/s40121-023-00862-6	Study protocol
REVIEWS OF INFECTIOUS DISEASES * VOL. 7, SUPPLEMENT 3 * JULY-AUGUST 1985	Part of combination given alone
10.1128/AAC.01411-17.	Part of combination given alone
10.3390/pharmaceutics15030804	Review
10.1007/s40121-020-00378-3	Review
10.1186/s12890-021-01472-z	Post-hoc analysis
10.1016/j.jemermed.2021.02.001	Post-hoc analysis
10.3390/antibiotics10121489	Pooled analysis
10.1097/MD.0000000000021223	Pooled analysis
10.1007/s40265-020-01443-4	Review
10.1136/bcr-2021- 243609	Case report with outcome present prior to intervention
10 .1128/AAC.01128-18	Safety not addressed
10.1128/AAC.02329-18.	PopPk analysis
10.1586/ERI.12.25	Review
10.1128/AAC.00071-20	Part of combination given alone
10.1093/cid/ciad097	Study protocol
