# Supplementary material for: Nephrotoxicity of New Antibiotics: A Systematic Review
Source: Toxics. 2025 Jul 19;13(7):606. doi: 10.3390/toxics13070606 (PMC12299473; doi:10.3390/toxics13070606)
Supplement: Supplementary file 1 [file toxics-13-00606-s001.zip › Supplementary Table S2 - Risk of bias assessment for clinical trials (Jadad scale).2-7-17.pdf]

**Supplementary Table S2.** Risk of bias assessment for clinical trials with the Jadad scale.

| **Author, year** | **Randomization (0-2)** | **Blinding (0-2)** | **Withdrawals (0-1)** | **Total Jadad score** | **Quality** |
|---|---|---|---|---|---|
| Carmeli, 2025 [139] | 2 | 0 | 1 | 3 | Moderate |
| ASSEMBLE study [54] | 2 | 0 | 1 | 3 | Moderate |
| Kaye, 2022 [56] | 2 | 2 | 1 | 5 | High |
| Bassetti, 2020 [140] | 2 | 0 | 1 | 3 | Moderate |
| Portsmouth, 2018 [58] | 2 | 2 | 1 | 5 | High |
| Wunderink, 2020 [59] | 2 | 2 | 1 | 5 | High |
| Awad, 2014 [73] | 2 | 0 | 1 | 3 | High |
| Bosheva, 2021 [74] | 2 | 0 | 1 | 3 | Moderate |
| Holland, 2023 [77] | 2 | 2 | 1 | 5 | High |
| Noel, 2008 [72] | 2 | 2 | 1 | 5 | High |
| Noel, 2008 [71] | 2 | 2 | 1 | 5 | High |
| Overcash, 2021 [76] | 2 | 2 | 1 | 5 | High |
| Zhao, 2022 [87] | 2 | 2 | 1 | 5 | High |
| O'Riordan, 2017 [90] | 2 | 1 | 1 | 4 | High |
| Taylor, 2018 [91] | 2 | 0 | 1 | 3 | Moderate |
| Wagenlehner, 2024 [96] | 2 | 2 | 1 | 5 | High |
| Ross, 2025 [97] | 2 | 0 | 1 | 3 | Moderate |
| Titov, 2020 [102] | 2 | 2 | 1 | 5 | High |
| Motsch, 2020 [101] | 2 | 2 | 1 | 5 | High |
| Sims, 2017 [98] | 2 | 1 | 1 | 4 | High |
| Alexander, 2019 [111] | 2 | 2 | 1 | 5 | High |
| File, 2019 [112] | 2 | 2 | 1 | 5 | High |
| Prince, 2013 [110] | 1 | 1 | 1 | 3 | Moderate |
| Connoly, 2018 [121] | 2 | 2 | 1 | 5 | High |
| Wagenlehner, 2019 [122] | 2 | 2 | 1 | 5 | High |
| Kaye, 2023 [125] | 2 | 0 | 1 | 3 | Moderate |
| Roberts, 2023 [103] | 2 | 2 | 1 | 5 | High |
