# Supplementary material for: Nephrotoxicity of New Antibiotics: A Systematic Review
Source: Toxics. 2025 Jul 19;13(7):606. doi: 10.3390/toxics13070606 (PMC12299473; doi:10.3390/toxics13070606)
Supplement: Supplementary file 1 [file toxics-13-00606-s001.zip › Supplementary Table S3 - Risk of bias assessment for observational studies (NOS scale).2-7-17.pdf]

Supplementary Table S3. Risk of bias assessment for observational studies with the Newcastle-Ottawa scale.

Author, year	Selection: Representativeness of Exposed Cohort	Selection: Selection of Non-Exposed Cohort	Selection: Ascertainment of Exposure	Selection: Outcome Not Present at Start	Comparability: Controlled for Confounders (1 star)	Comparability: Additional Control (1 star)	Outcome: Assessment of Outcome	Outcome: Follow-Up Long Enough	Outcome: Adequacy of Follow-Up	Total Stars (out of 9)	Quality
Campogiani, 2023 [64]	1	0	1	1	0	0	1	0	0	4	Moderate
Clancy, 2024 [66]	1	0	1	1	0	0	1	0	0	4	Moderate
Karruli, 2023 [65]	1	0	1	1	0	0	1	1	1	6	Moderate
Oliva, 2024 [67]	1	1	1	1	1	1	1	0	0	7	High
Falcone, 2022 [63]	1	1	1	1	1	1	1	1	1	9	High
Crapis, 2020 [78]	1	0	1	1	0	0	1	0	0	4	Moderate
Durante-Mangoni, 2020 [80]	0	0	1	1	0	0	1	0	0	3	Low
Membrillo De Novales, 2025 [82]	1	1	1	1	1	1	1	1	1	9	High
Zampino, 2023 [81]	1	1	1	1	1	1	1	0	0	7	High
Caniff, 2025 [104]	0	0	1	1	1	1	1	0	0	5	Moderate

Shimada, 2024 [109]	1	0	1	1	1	0	1	0	0	5	Moderate
Mehta, 2022 [116]	1	0	1	1	0	0	1	0	0	4	Moderate
Mehta, 2022 [117]	1	0	1	1	1	1	1	0	0	6	Moderate
Saseedharan, 2024 [118]	1	0	1	1	1	1	1	0	0	6	Moderate
Telkhade, 2024 [119]	1	0	1	1	0	0	1	0	0	4	Moderate
Arnés García, 2023 [79]	1	1	1	1	1	1	1	0	0	7	High
