# Supplementary material for: Nephrotoxicity of New Antibiotics: A Systematic Review
Source: Toxics. 2025 Jul 19;13(7):606. doi: 10.3390/toxics13070606 (PMC12299473; doi:10.3390/toxics13070606)
Supplement: Supplementary file 1 [file toxics-13-00606-s001.zip › Supplementary Table S4 - Risk of bias assessment for non-randomized clinical trials (ROBINS-I v2).2-7-17.pdf]

Supplementary Table S4. Risk of bias assessment for non-randomized clinical trials (ROBINS-I V2).

Author, year	Confounding (baseline gfr, diabetes, hypertension, medications)	Selection Bias	Intervention Classification	Deviations from Intervention	Missing Data	Outcome Measurement	Reporting Bias	Overall Risk of Bias
Overcash, 2020 [93]	Moderate	Low	Low	Low	Low	Low	Low	Moderate
Iwanaga, 2025 [108]	High	Low	Low	Low	Low	Low	Low	High
Takazono, 2024 [107]	Moderate	Low	Low	Low	Low	Low	Low	Moderate
Bradley, 2025 [61]	Low	Low	Low	Low	Low	Low	Low	Low
Cornely, 2020 [52]	Low	Low	Low	Low	Low	Low	Low	Low
