# Supplementary material for: Nephrotoxicity of New Antibiotics: A Systematic Review
Source: Toxics. 2025 Jul 19;13(7):606. doi: 10.3390/toxics13070606 (PMC12299473; doi:10.3390/toxics13070606)
Supplement: Supplementary file 1 [file toxics-13-00606-s001.zip › Supplementary Table S5 - Studies not assessed for risk of bias with comment.2-7-17.pdf]

Supplementary Table S5. Studies not assessed for risk of bias.

**Author, year**	**Comment**
Das, 2020 [55]	Phase 1 study
Katsube, 2017 [62]	Phase 1 study
Saisho, 2018 [57]	Phase 1 study
Cipko, 2021 [68]	Case report
Li, 2021 [75]	Phase 1 study
Li, 2023 [89]	Phase 1 study
Schmitt-Hoffman, 2004 [70]	Phase 1 study
Eckburg, 2017 [84]	Phase 1 study
Wu, 2018 [85]	Phase 1 study
Wu, 2019 [86]	Phase 1 study
Yang, 2023 [88]	Phase 1 study
Barth, 2023 [95]	Phase 1 study
Hossain, 2020 [92]	Phase 1 study
Tiffany, 2022 [94]	Phase 1 study
Machuca, 2024 [105]	Case series
Rhee, 2018 [99]	Phase 1 study
Totsuka, 2019 [106]	Phase 1 study
Hu, 2023 [115]	Phase 1 study
Wicha, 2019 [113]	Phase 1&2 study
Wicha, 2021 [114]	Phase 1 study
Cass, 2011 [120]	Phase 1 study
Gall, 2019 [123]	Phase 1 study
O'Donnell, 2019 [124]	Phase 1 study
Zhanel, 2021 [83]	Cross-sectional registry study
Sollima, 2020 [69]	Case series
Bhagunde, 2020 [100]	Phase 1 study
